# Chitosan: A Natural Biopolymer with a Wide and Varied Range of Applications

**DOI:** 10.3390/molecules25173981

**Published:** 2020-09-01

**Authors:** Carmen P. Jiménez-Gómez, Juan Antonio Cecilia

**Affiliations:** Departamento de Química Inorgánica, Cristalografía y Mineralogía (Unidad Asociada al ICP-CSIC), Facultad de Ciencias, Universidad de Málaga, Campus de Teatinos, 29071 Malaga, Spain; carmenpjg@uma.es

**Keywords:** chitin, chitosan, biomaterial, adsorbent, antioxidant, high added value product

## Abstract

Although chitin is of the most available biopolymers on Earth its uses and applications are limited due to its low solubility. The deacetylation of chitin leads to chitosan. This biopolymer, composed of randomly distributed β-(1-4)-linked D-units, has better physicochemical properties due to the facts that it is possible to dissolve this biopolymer under acidic conditions, it can adopt several conformations or structures and it can be functionalized with a wide range of functional groups to modulate its superficial composition to a specific application. Chitosan is considered a highly biocompatible biopolymer due to its biodegradability, bioadhesivity and bioactivity in such a way this biopolymer displays a wide range of applications. Thus, chitosan is a promising biopolymer for numerous applications in the biomedical field (skin, bone, tissue engineering, artificial kidneys, nerves, livers, wound healing). This biopolymer is also employed to trap both organic compounds and dyes or for the selective separation of binary mixtures. In addition, chitosan can also be used as catalyst or can be used as starting molecule to obtain high added value products. Considering these premises, this review is focused on the structure and modification of chitosan as well as its uses and applications.

## 1. Introduction

**Figure 1:**
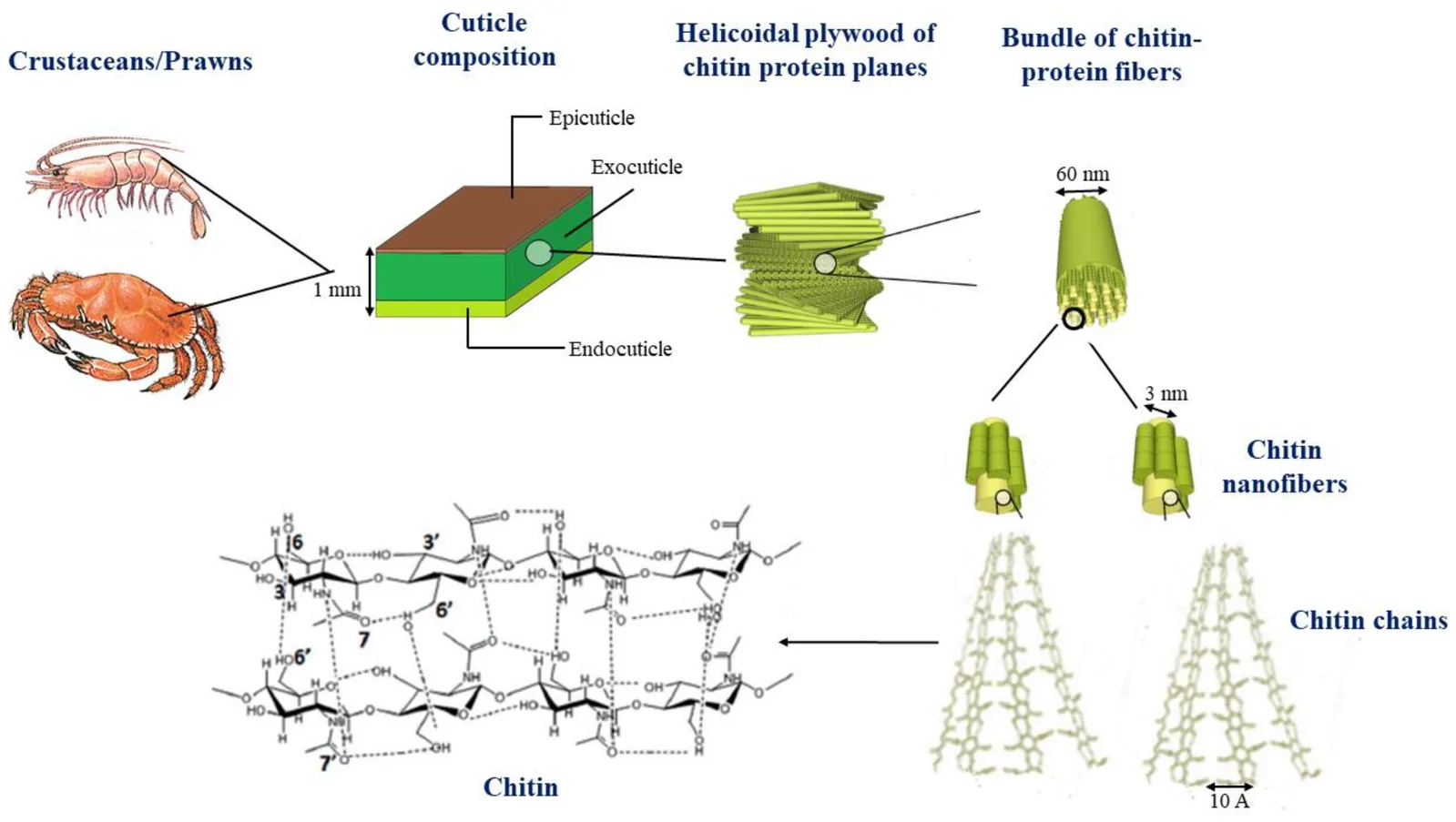
Structural composition and arrangement of chitin in the shell of crustaceans.

Chitin is considered the second most abundant polysaccharide (after cellulose) on Earth, being first described by Henri Braconnot in 1811. It appears in Nature as ordered macrofibrils in the exoskeleton of mollusks and crustaceans, as well as in fungi and insect cuticles [1] (Table 1 and Figure 1). Its natural abundance allows obtaining more than 1000 tons every year, of which about 70% comes from marine species [2].

From a chemical viewpoint, chitin is a poly(β-(1-4)-*N*-acetyl-d-glucosamine) with β(1→4) linkages [1], and it is considered to have a cellulose-like structure, where the hydroxyl group in the C2 position has been replaced by an acetamido group (Figure 1). Depending on the orientation of polysaccharide chains, chitin displays three polymorphs, denoted as α, β and γ [3]. Among them, the α-type is the most abundant in shellfish shells. This polymorph has an antiparallel arrangement, where each chain strongly interacts with the adjacent through hydrogen bonds, which provide high thermochemical stability, as well as high insolubility [4,5]. Thus, chitin is a highly insoluble polymer, making it poorly biodegradable. Thus, only the action of chinitanase enzymes, widely distributed in nature, can degrade chitin [6]. This low solubility in water and most of organic solvents has limited its uses and applications.

**Figure 2:**
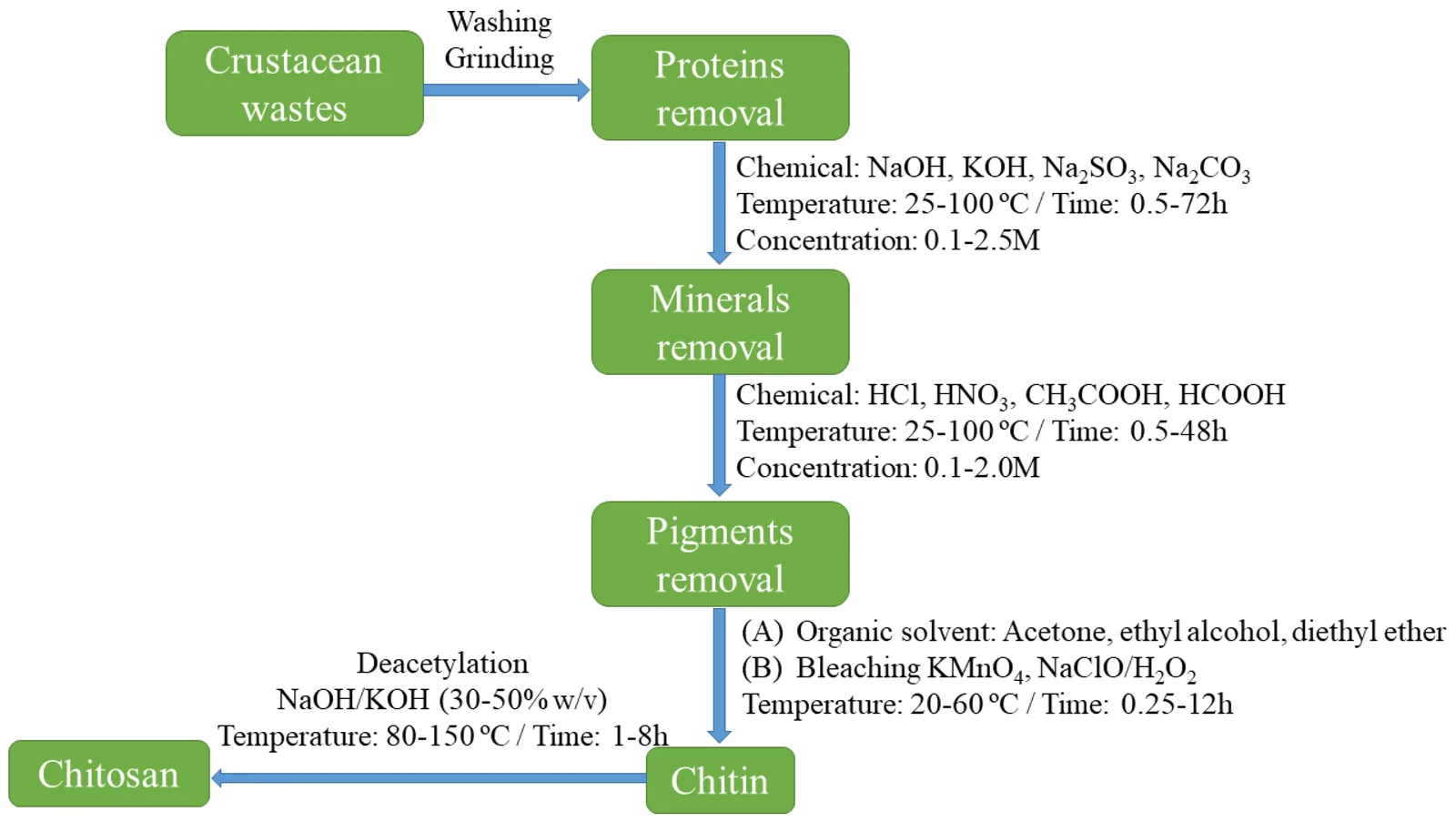
Purification processes of crustacean wastes.

Nowadays, there is a well-established protocol for the chitin extraction from shellfish wastes in industry, which includes the steps of demineralization, deproteinization and decolorization [7,8]. The deproteinization is carried out by an alkaline treatment, whereby lipids and proteins are hydrolyzed. The demineralization stage is generally performed in the presence of acids, whereas the decolorization requires an oxidative treatment (Figure 2) [9,10]. Finally, chitin, in turn, can be deacetylated by a basic treatment to give rise to chitosan, which is a soluble polymer in acid aqueous medium. Depending on the production method and species used, the degree of deacetylation ranges from 56 to 99%, but at least 85% deacetylation is required for a good solubility of chitosan [11]. Table 2 compiles most of methods employed to quantify the deacetylation degree and the molecular weight of the chitosan obtained.

## 2. Chitosan

Chitosan was discovered by Rouget in 1859 after heating chitin in an alkaline medium [26]. Several years later, Hoppe-Seyer called this material chitosan, although its chemical structure was not elucidated until 1950 [27].

**Table 3:** Main chemical properties of chitosan, according the information reported in [28].

Linear aminopolysaccharide with a high nitrogen content
Rigid d-glucosamine structure: hydrophilicity, crystallinity
Weak base (pK_a_: 6.3). Deprotonated amino group can act as strong nucleophile
Enable to form intermolecular hydrogen bonds: high viscosity
Existence of reactive groups for chemical activation and cross-linking
Insoluble in water and organic solvents, but soluble in dilute aqueous acid solutions.
It forms salts with organic and inorganic acids
Complexing and chelating properties
Ionic conductivity
Polyelectrolytes (at acid pH)
Cationic biopolymer with high charge density (one positive charge per glucosamine residue)
Flocculating agent (interacts with negatively charged molecules)
Entrapment and adsorption properties (filtration and separation)
Film-forming ability (adhesive materials for isolation of biomolecules)
Biological properties (biocompatibility) bioadhesivity bioactivity non-toxic biodegradable adsorbable antimicrobial activity (fungi, bacterial, viruses) antiacid, antiulcer and antitumoral properties blood anticoagulants hypolipidermic activity

**Figure 3:**
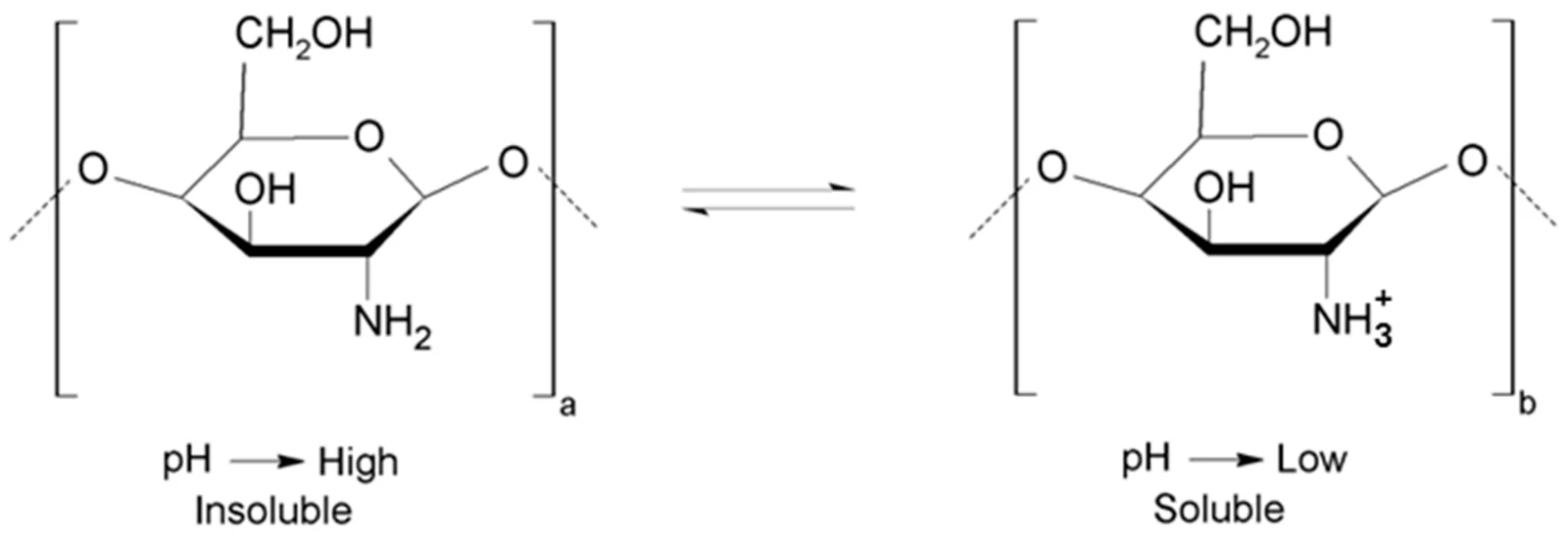
Chemical structure of chitosan as a function of pH. Insoluble (pH > 6) and soluble (pH < 6).

In recent years, chitosan-based materials have been developed due to their particular chemical properties, which provide it a wide range of applications as indicated in Table 3. Generally, chitosan is highly soluble in acid solution (mainly below pH = 6.0), being a weak base (pKa = 6.3) due to the presence of amine groups. At low pH values, the amine groups are positively charged due to protonation, so chitosan can be a water-soluble cationic polyelectrolyte. However, when the pH increases above 6, the amine groups of chitosan residues are deprotonated and the biopolymer loses its charge leading to an insoluble polymer (Figure 3) [29].

### 2.1. Modification of Chitosan by Functionalization

The presence of amine and hydroxyl groups provides interesting applications to chitosan, since these can be modified to improve certain properties of this biopolymer. Among some chemical processes to improve their properties, cross-linking, graft copolymerization, carboxymethylation, etherification, and esterification must be highlighted as the main strategies to functionalize the chitosan structure [30].

#### 2.1.1. Cross-Linking/Hydrophobic Interactions

A hydrogel is defined as a polymeric structure where the chains are linked through non-covalent and/or covalent bonds to form a tridimensional network. These structures possess the ability to retain large amounts of water, causing the swelling of the structure. Chitosan is a biopolymer that can form hydrogels as a consequence of small modifications of ionic strength or pH.

**Figure 4:**
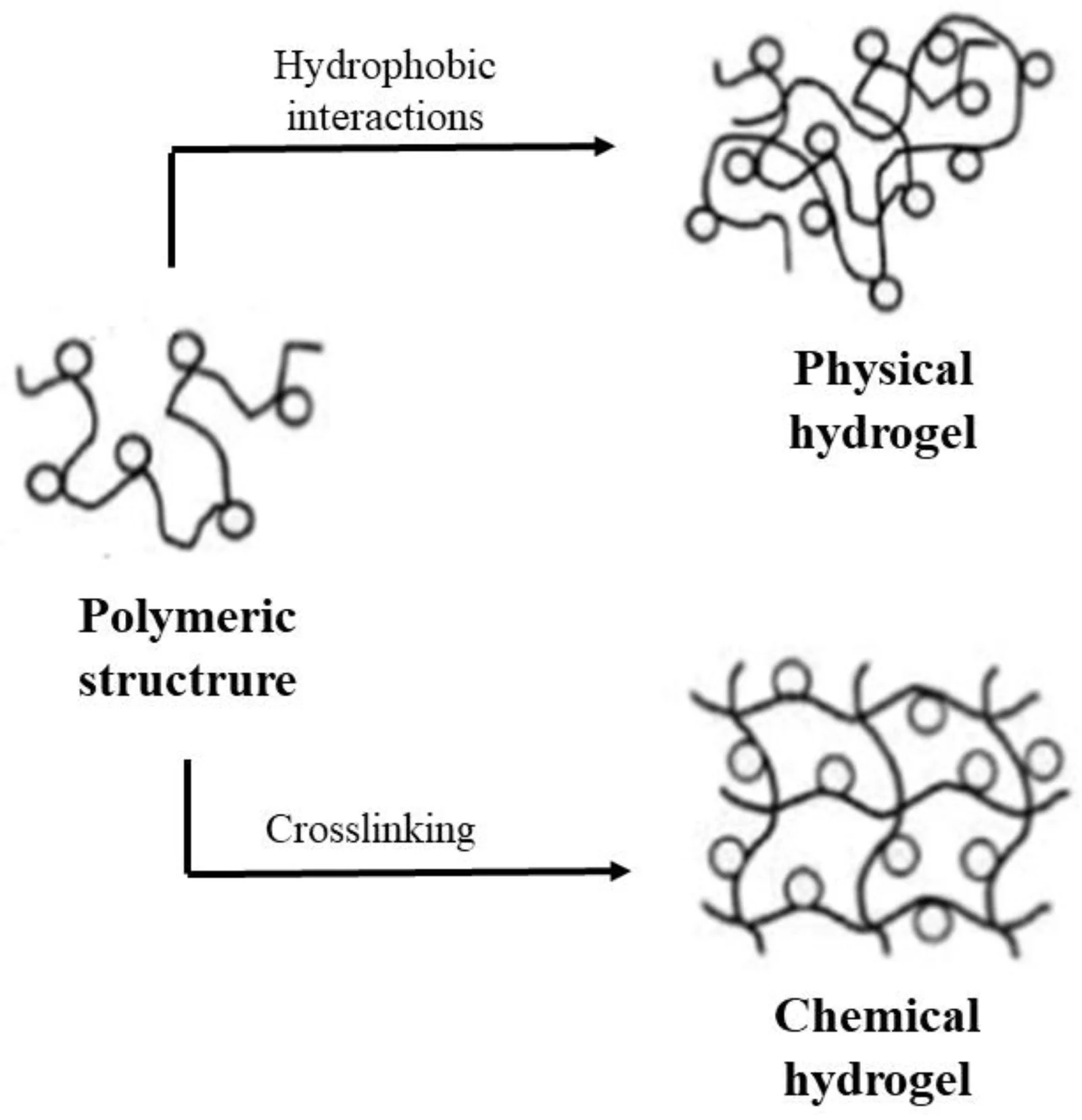
Chitosan hydrogels obtained by hydrophobic or crosslinking interactions.

As was previously indicated, the amine groups of chitosan are protonated in an acidic medium. This fact causes electrostatic repulsions, which promote the swelling of the chitosan structure [31]. The formation of hydrogels takes place mainly by electrostatic interactions of the hydroxyl groups located in C-3 and C-6 positions and the amine group located in the C-2 position of the monomers. Thus, chitosan tends to form cross-linked tridimensional structures with dialdehydes, such as glyoxal or glutaraldehyde, which are used, for example, to develop membranes with proton conductivity, which have potential use in fuel cells [32]. On the other hand, glutaric and adipic acids were also employed in the synthesis of biocompatible chitosan hydrogels [33,34]. In all cases, it has been reported that the degree of cross-linking is directly related to the properties of hydrogels, such as swelling degree, mechanical strength or pore size, among others (Figure 4). Chitosan hydrogels can also be used as controlled release systems, since they are able to maintain a constant drug concentration for a prolonged time in a particular environment [35,36]. As chitosan is a biocompatible polymer, these hydrogels have been used to prepare biodegradable sutures, hemodialysis membranes, healing of wound and burns, cells or immobilizing enzymes [2].

#### 2.1.2. Graft Copolymerization

**Figure 5:**
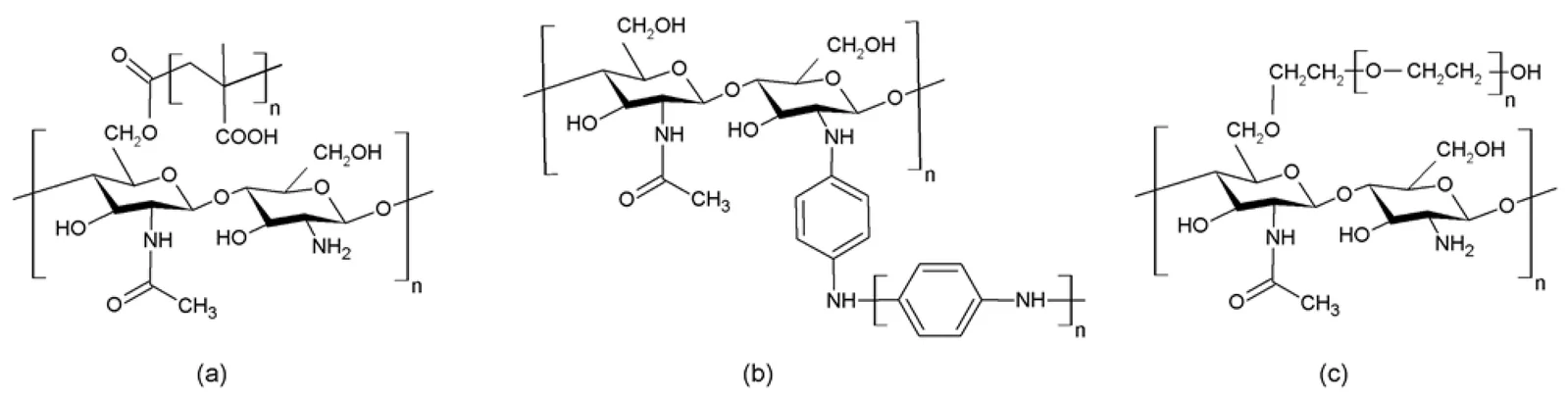
Some examples of chitosan graft copolymers: (**a**) chitosan/PMMA, (**b**) chitosan/PANI and (**c**) chitosan/PEG.

The graft copolymerization of synthetic polymers with chitosan is of great interest in several fields. It has been reported that a chitosan/methacrylate composite can be synthesized using ammonium persulfate (APS) as initiator, obtaining a copolymer whose solubility is much higher than that reported for their respective chitosan hydrogels (Figure 5A) [37]. Other authors have pointed out that the copolymerization of chitosan/aniline using, APS initiator leads to films with protonic conductivity (Figure 5B) [38]. The use of polyethylene glycol as a copolymer of chitosan has been widely studied (Figure 5C), being considered as suitable graft-forming polymers due to its good solubility both in H_2_O and inorganic solvents, high biodegradability and biocompatibility or low toxicity [39].

#### 2.1.3. Carboxymethylation

As was indicated in the previous sections, one of main disadvantages of chitosan is its low solubility. Carboxymethylation is an alternative to improve its solubility in aqueous media. This process takes place by the dispersion of chitosan in 2-propanol in basic medium.

**Figure 6:**
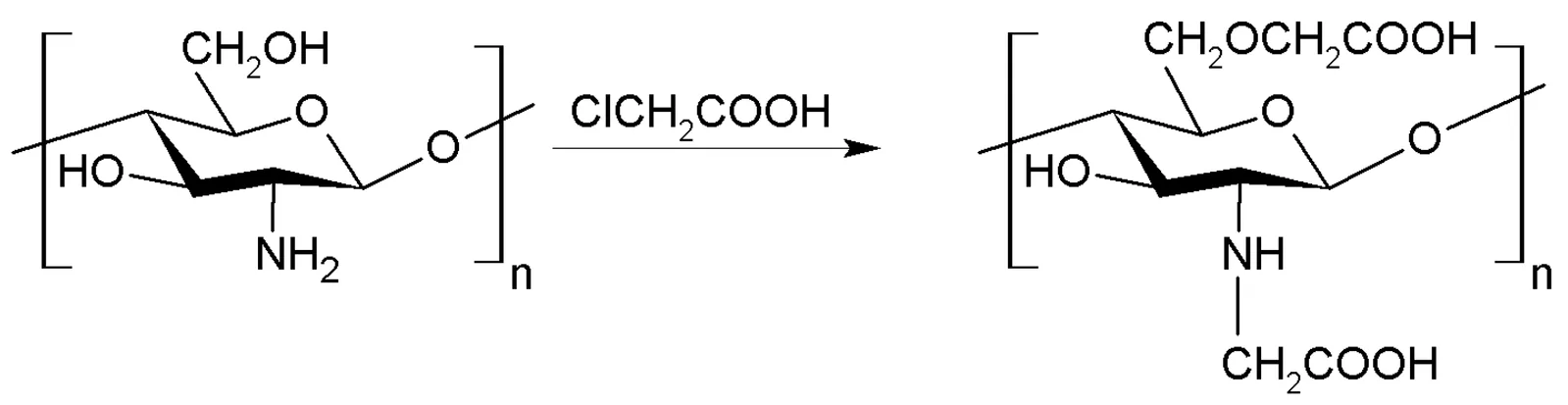
Reaction of chitosan O- and N- carboxymethylation.

In the next step, a 2-propanol/monochloroacetic acid mixture is added to the first suspension [40]. It must be considered that *O*- and *N*-carboxymethylation may occur simultaneously, although several parameters can be controlled in such a way the reaction takes place through one of them (Figure 6). The final product (chitosan-carboxymethyl) is an amphoteric polymer whose solubility depends on pH.

#### 2.1.4. Etherification

Chitosan was also grafted with propylene epoxide under basic conditions to form a hydroxypropylchitosan composite. Other authors have reported that the etherification reaction is carried out to improve the its solubility in organic solvents and water, charge, hydrophilicity and ability to interact with other substances [41]. Chitosan ethers can be applied in pharmaceutical, biomedical, adsorption, and environmental fields [42,43]. From this etherification process, it has been noted that the hydroxypropyl chitosan copolymer improves the antimicrobial applications, showing good inhibition effect against *E. coli* and *S. aureus* [37]. On the other hand, trimethylchitosan displays high solubility in water over a wide pH range in such a way that it can form stable ionic complexes with DNA and is therefore employed in DNA delivery [44]. It has been also reported that cyanoethyl chitosan improves solubility in organic solvents and can be used in dialysis, filtration and insulating papers [45] while hydroxyethyl chitosan and hydroxypropyl chitosan membranes improves the ionic conductivity about one order of magnitude in comparison to the pristine chitosan membrane [41].

#### 2.1.5. Esterification

**Figure 7:**
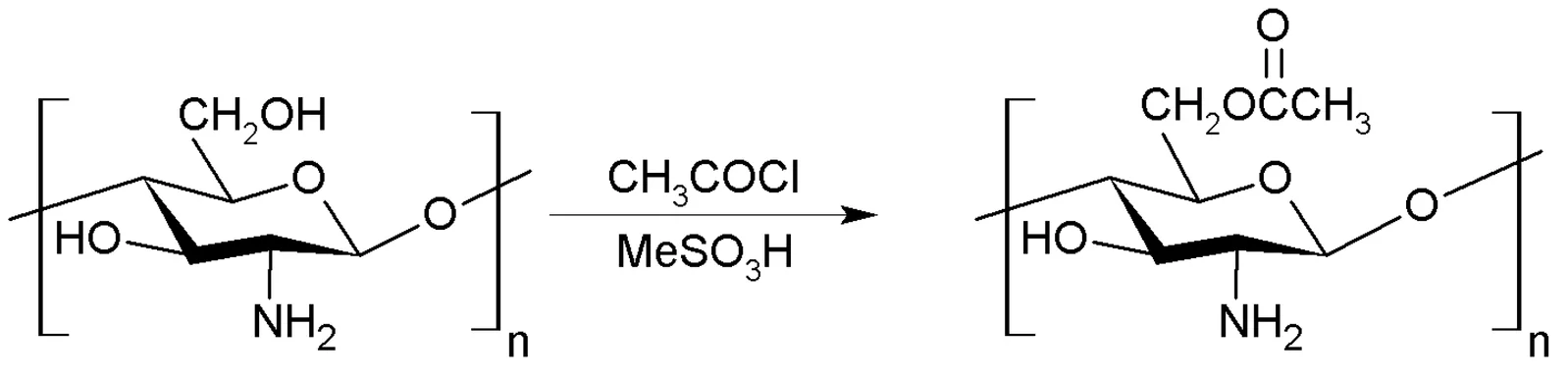
*O*-acylation of chitosan.

Another alternative to improve certain properties of chitosan is to carry out esterification reactions with the chitosan molecule. Previous research have stablished the synthesis of *N*,*O*-acyl chitosan with acetyl chloride by using MeSO_3_H as solvent (Figure 7). This reaction can lead to *O*- and *N*-acetylation although *O*-acetylated chitosan is the main product. In any case, the acetylation of chitosan substantially improves its antifungal activity [46,47].

#### 2.1.6. Phosphorilation

**Figure 8:**
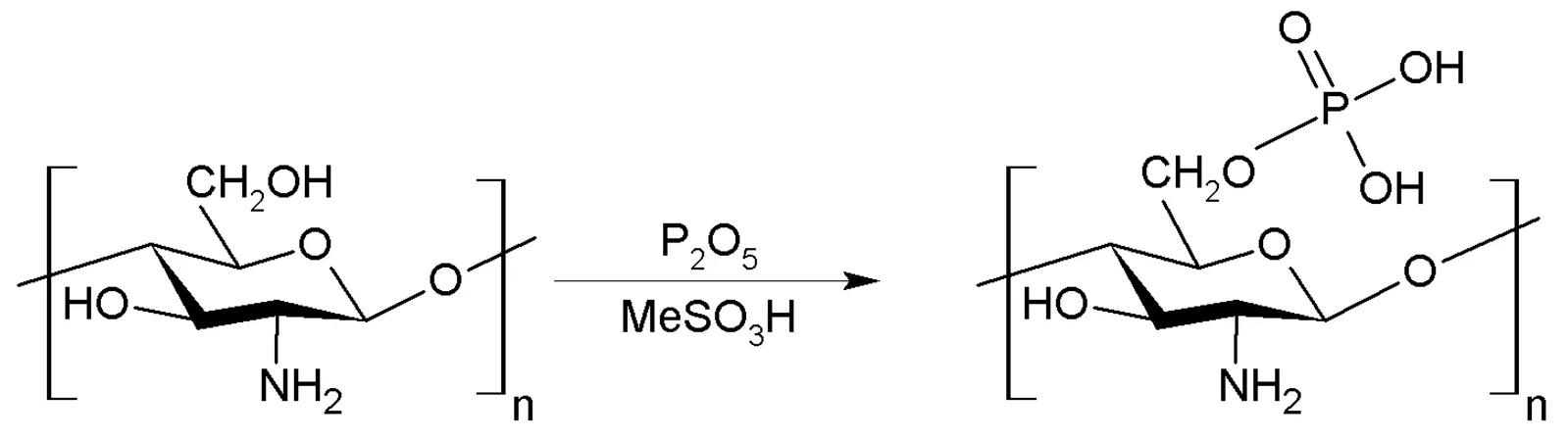
Phosphorylation of chitosan using P_2_O_5_.

The phosphorylation of chitosan modifies their biological and chemical properties due to this treatment improves its bactericidal and osteoinductive properties. Generally, the phosphorylation of chitosan takes place in the C-3 and C-6 positions (Figure 8). Phosphorylated chitosan can be obtained by heating chitosan with phosphoric acid using *N*,*N*-dimethylformamide (DMF) as solvent. Another alternative to synthesize phosphorylated chitosan is through the reaction of chitosan with phosphorous pentoxide in the presence of methanesulphonic acid [48].

In its anionic form, phosphonic chitosan can interact with some amphoteric cations such as Ca^2+^, Cu^2+^, Cd^2+^ or Zn^2+^ [49]. This complexation process protects the surface of metals againts corrosion processes [50]. On the other hand, these phosphoriled derivatives can also be grafted with alkyl groups to improve their amphiphilic properties, being used in the cosmetic field [51].

#### 2.1.7. Sulphatation

Both *O*-sulfated derivative [52] and *N*-sulfated chitosan [53] are of interest in biomedical applications as anticoagulants. In this sense, it has been reported that the degreed of sulfate substitution influence in the anticoagulant activity of chitosan, obtaining a similar anticoagulant activity to that reported for the heparin due its structural similarity [54]. On the other hand, sulfated chitosans are potent scavengers of free radical ions, including hydroxyl and superoxide ions [55].

#### 2.1.8. Guanidinylation/Biguanidinylation

Both guanidinylation and biguanidinylation of chitosan by grafting are effective and easy way to prepare composites which interact strongly with plasmic DNA in such a way it enhances the gene delivery [56]. In the same way, other authors reported that the guanidinylation of chitosan also has an excellent antibacterial activity although the grafting reaction between guanidine and chitosan requires high temperature [57,58]. More recently, other authors established that the microwave assisted preparation of antimicrobial chitosan with guanidine oligomers diminishes the temperature of the grafting reaction [59]. The obtained composite also showed a significant increases of the antimicrobial activity in comparison to the raw chitosan [59]. In the same way, the guanidinylation reaction of chitosan with dicyandiamide to form chitosan biguanidine hydrochloride has also reported excellent antibacterial and antifungal applications [60].

## 3. Applications of Chitosan

Taking into account that chitosan can be modified to improve some of its physical, chemical or biological properties, these chitosan-based materials have been highly tested in a wide range of applications. Some of the main applications will be highlighted in Table 4 and the next sections.

### 3.1. Uses in Pharmacy and Medicine

As was previously described, chitosan is a biopolymer with interesting biomedical applications due to its low toxicity, biodegradability and biocompatibility. This biopolymer is prone to be degraded by enzymatic hydrolysis with lysozyme, which is a proteolytic enzyme that is present in the tissues of all humans. In the same way, lipase present in the human gastric, pancreatic fluid or saliva is also able to degrade chitosan. In all cases, the obtained product were non-toxic [61].

The contact of the chitosan amine groups with the acid groups of the blood cells leads to the formation of clots as a consequence of thrombogenic and/or hemolytical response. The mechanism of chitosan-blood interaction starts with an adsorption of the plasma on the surface of chitosan, followed by the adhesion and activation of platelets that form thrombus [62]. It has been reported in the literature that sulfatation of the hydroxyl or amine groups of chitosan improves its behavior of the raw chitosan as anticoagulant, antioxidant, antimicrobian and hemagglutination inhibition [63].

Chitosan displays excellent applications as a hypocholesterolemic and hypolipidemic agent, in such a way chitosan can reduce the risk of cardiovascular diseases. In addition, chitosan shows interesting antimicrobial and antioxidant properties. All these properties has led to this biopolymer and its derivatives have applications beyond medicine since it can also be applied biodegradable sponges, surgical sutures, membranes, microspheres, tablets, delivery drugs [64,65].

### 3.2. Biomaterial

Chitosan displays a wide range of applications as a biomaterial due to its good behavior in the human body since it has been reported that this biopolymer displays antimicrobial activity, bioactivity, chemotactic action, immunostimulaion, enzymatic biodegradability, mucoadhesion, or epithelial permeability, favoring its adhesion with different types of cells [66]. Due to this excellent behavior, on one hand, chitosan together with chitin have been used in treatment of wounds, burns and ulcers due to its haemostatic characteristics and hastening wound healing effect. On the other hand, chitosan has been also used in tissue regeneration and restoration due to its biodegradability and cell affinity [67].

### 3.3. Tissue Engineering

Several materials are developed according to the wound type and healing mode. These biocomposites may contain synthetic polymers like polyurethane rubber and natural polymers such as collagen, chitosan, gelatin and alginate. Bioactive dressings are reported to have better quality as compared to synthetic dressings (Table 5).

Tissue engineering involves the use of living cells, which are generally manipulated from their extracellular environment to synthesize tissue that can be implanted into the body [68,69]. Generally, the tissue engineering is employed to repair, maintain, replace or enhance the function of a specific tissue or organ [70]. Chitosan has been used as polymer scaffold in tissue engineering due to the fact these structures display some properties such as high porosity, biodegradability, structural integrity and non-toxic to cell as well promoting the interaction with the cells to favor its adhesion and it should also encourage cell function [71]. The materials obtained from tissue engineering have shown excellent results as cartilage membranes [72], nerve [72], bone [73] and tracheal tissue [74].

Bones consist of mainly hydroxyapatite (Ca_10_(PO_4_)_6_(OH)_2_), together with other components such as collagen, keratin sulfate, chondroitin sulfate and lipids. The treatment of broken bone or damage can be carried out using a biodegradable compound, which is used as temporary skeleton to substitute the lost bones or the defective sites. Then, this bone support is progressively degraded and replaced by the new bone tissue without having any adverse effect on health throughout the treatment. Bioactive ceramics are chemically similar to natural bone, which allows osteogenesis to occur and can provide a bony contact or bonds with the host bone; however, the limitations of these bioceramic compounds are related to its low biodegradability and brittleness [75]. In order to minimize these drawbacks, several biopolymers such as chitosan have been studied in bone tissue engineering due to its capacity to promote growth and mineral rich matrix deposition by osteoblasts in culture [76]. Thus, the synthesis of composite/hydroxyapatite composites have shown interesting applications in the bone reparation since the hydroxyapatite structure strengthened the chitosan matrix and adjust the release burst effect [65].

**Figure 9:**
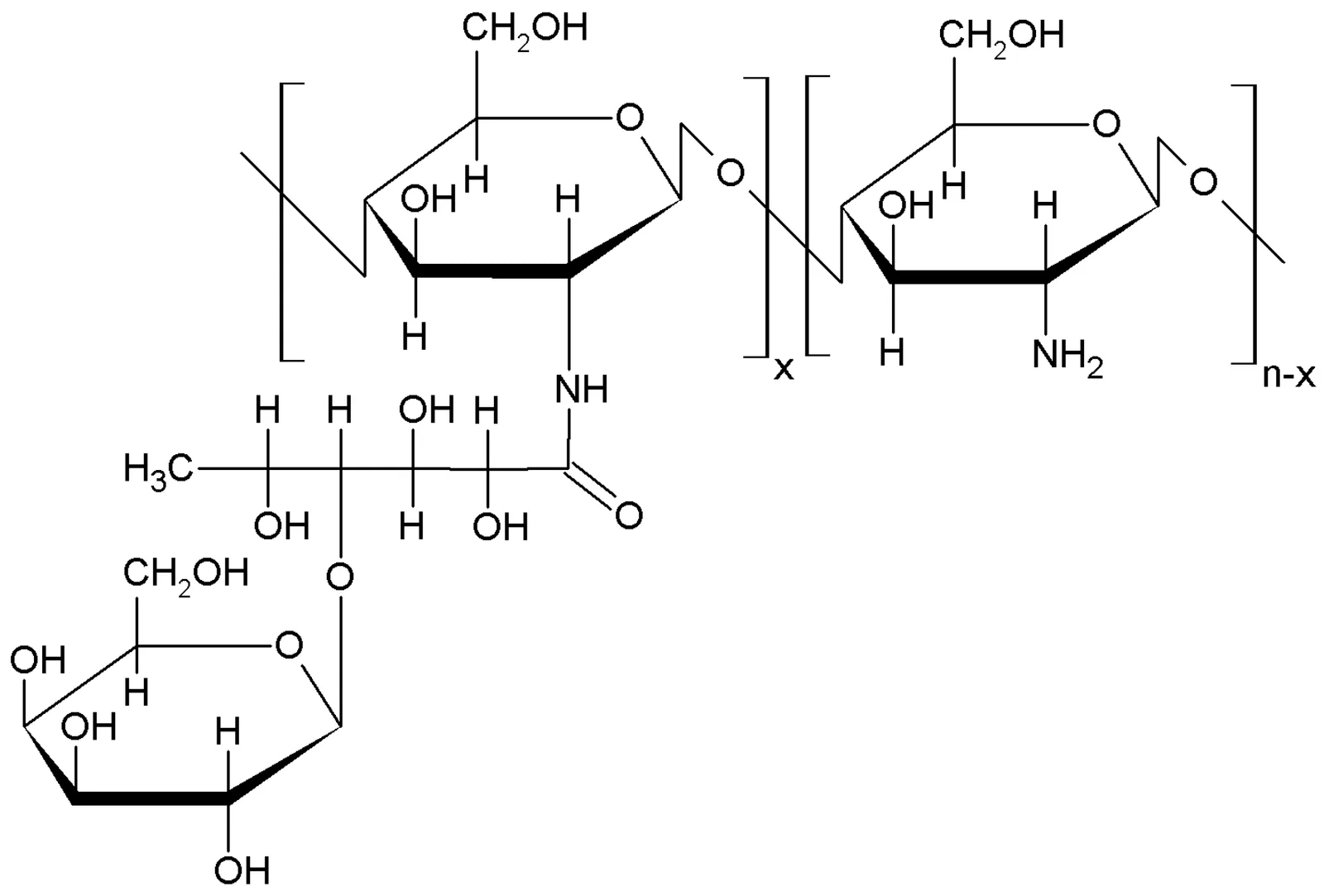
Chemical structure of galactosylated chitosan.

Articular cartilage is a connective tissue whose principal functions is to facilitate the lubrication of the articulations and the transmission of loads to diminish the friction coefficient. Articular cartilage consists of isolated articular chondrocytes as well as their precursor cells that may be expanded in vitro and then seeded into a biocompatible matrix or scaffold for cultivation and subsequent implantation into a joint. The selection of a suitable biomaterial is a key factor for the successful repair of cartilage [77]. In this context, it has been reported that cartilage-specific extracellular matrix components such as collagen and glycosaminoglycans can play an important role in the regulation of the chondrodrocytic phenotype in supporting chondrogenesis both in vivo and in vitro (Figure 9). As chitosan displays a similar structure to glycosaminoglycans, it is a biopolymer with potential to be used as scaffolding material in articular cartilage engineering [78]. Thus, chitosan-chondroitin sulphate membranes and collagen-glycosamino glycans-chitosan have shown good results in the repair of cartilage and human skin [78,79]. Other authors prepared chitosan-based scaffolds by combining it with alginate. In this work, chitosan was modified with lactobionic acid to produce galactosylated chitosan, which was added to cross-linked alginated gel and finally freeze-drying lyophilization. These materials display pores whose size depends on the freeze-drying treatment, the molecular weight and the proportion of galactosylated chitosan. These authors carried out a study on human endothelial cells on chitosan, which had cell-adhesive peptides photochemically grafted onto their surfaces.

Nerve injuries are one of most complicated to repair due to the fact neurons have scant ability to undergo cell division. All attempts to repair fibrous nerves have been focused on the regenerating nerve fiber into the proper endoneurial tubes. Several materials have been proposed for generating artificial tubes to repair nervous fibers [80]. These materials must be biocompatible and biodegradable as well as provide a modulated cellular, structural and molecular framework. Considering the required properties, chitosan is considered as potential biomaterial to repair nerve injuries due to its special properties such as antibacterial activity, biodegradability, biocompatibility and antitumoral activity [81]. It has been reported that chitosan membranes can grow and repair neurons of the peripheral nervous system [82]. In the same way, other authors have pointed out that chitosan fiber can also reinforce the adhesion, migration and proliferation of Schwann cells, which provide a similar effect for regenerating axons in the nervous system [83].

**Figure 10:**
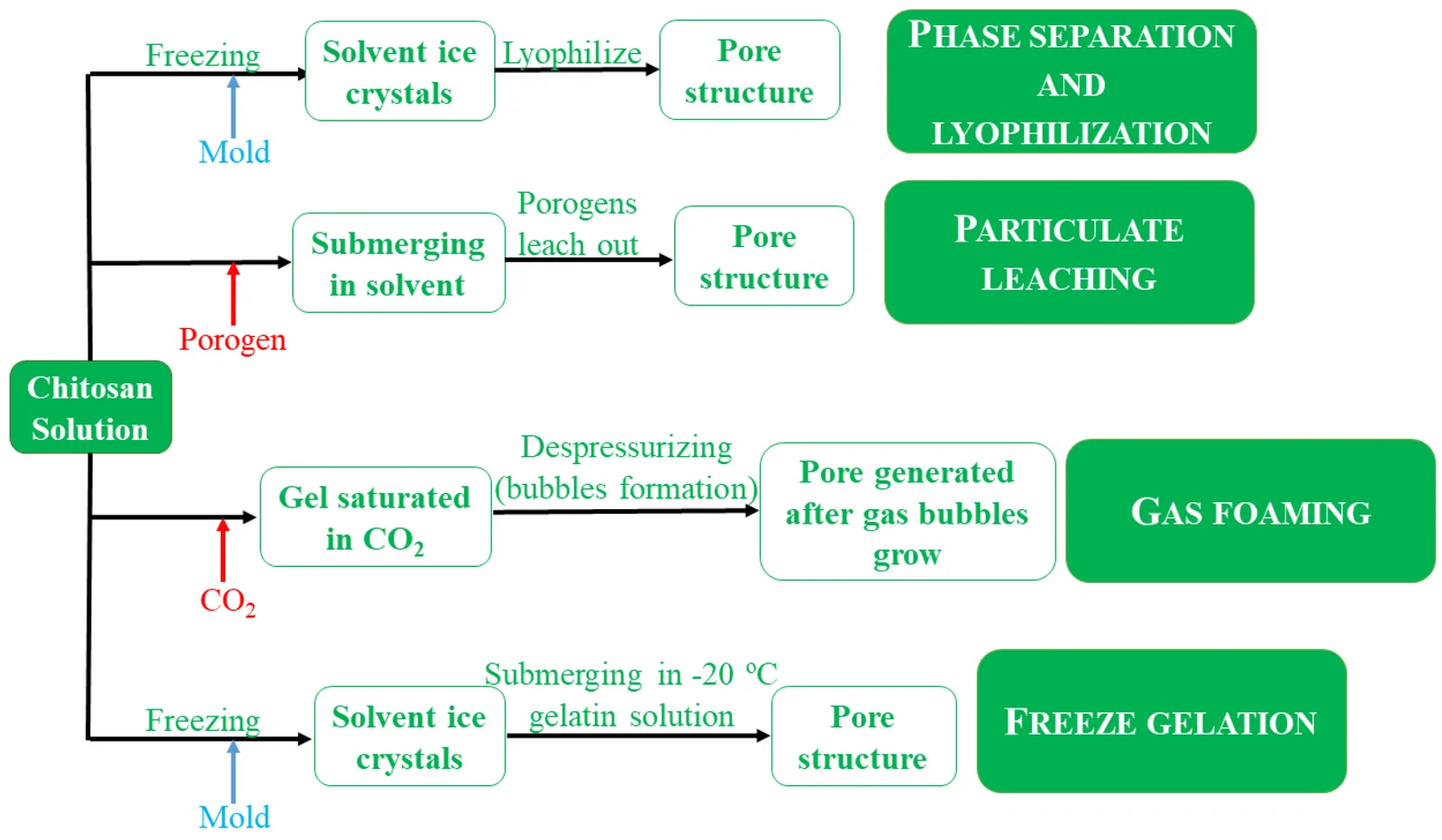
Different methodologies to synthesize three-dimensional chitosan scaffolds.

Several methods to generate three-dimensional chitosan scaffolds for initial cell attachment and the formation of the subsequent tissue It has been reported (Figure 10). Among them, the following can be highlighted:

- Phase separation and lyophilization. Firstly, a chitosan solution is introduced into a mold and then a freezing step makes it ready for phase separation with acetic acid as solvent and chitosan acetate salt.
- Particulate leaching techniques. A porogen, usually gelatin, is mixed with a chitosan solution prior to phase separation and lyophilization steps. When is submerged in a solvent, the scaffold is formed through porogen leaching. This fact implies that the obtained scaffolds can have an additional porosity.
- Gas foaming. A chitosan solution contains a cross-linked, mainly glutaraldehyde, which is saturated with CO_2_ under high pressure, favoring the cross-linking. When the system is depressurized, the thermodymamic instability leads to nucleation and gas bubble growth. The porosity is formed by the bubbled space of the polymer solution).
- Freeze gelation. The obtained scaffolds is placed in a gelation solution of NaOH and ethanol below the chitosan freezing temperature. Then, the gel is air-dried to remove the residual liquid.

### 3.4. Wounds and Burns

The healing of wound and burns is a biological process related with growth and tissue regeneration. The wound healing process has five important steps [84]:

- Homeostasis
- Inflammation
- Migration
- Proliferation
- Maturation

Several studies have reported the use of chitosan membranes to facilitate wound healing with potential application in patients with severe burns or wounds. It has been reported that the use of polyvinyl alcohol-chitosan composite membranes accelerates the mechanical properties of the obtained composite. In the same way, glycerol-oleic acid-chitosan composites have shown to be cyto-compatibility, biocompatible, bioadsorbable and provide sustained drug release. Chitosan-based composites have great importance in the field of wound care to avoid complications (infections or poor wound healing); thus, Ag-chitosan has been used as an antibacterial agent [84,85]. Similarly, Cu-chitosan or ZnO-chitosan have also shown good wound healing properties since these composites favor collagen deposition, fibroblast proliferation and re-epithelialization [86,87].

Blazevic et al. synthesized chitosan-lecithin nanoparticles for release of melatonin to improve the wound healing, obtaining good results for wound epithelialization [88]. Archana et al. have synthesized a composite formed by chitosan/TiO_2_/N-vinylpyrrolidone. This composite had excellent antimicrobial efficiency against several pathogenic bacteria as a consequence of a good biocompatibility, antibacterial ability, wound appearance, elevated swelling properties and hydrophilic nature [89].

The treatment of the skin after burns can also be carried out using chitosan since this biopolymer forms resistant films which can be directly deposited on the burned skin by the application of an aqueous solution of chitosan acetate [90]. In addition, chitosan can also facilitate the oxygen permeability, which is essential for the healing of burns. Moreover, chitosan film has the ability to adsorb water and then it can be automatically degraded by body enzymes [91].

Those individuals who have suffered extensive losses skin by burns have a high risk of dying from fluid loss as well as massive infections. Several studies have reported that chitosan/ glycoaminoglycan-based composites have potential to be used in a skin replacement. Thus, some authors have developed a wound covering material from polyelecrolyte complexes of chitosan and sulfonated chitosan, which accelerates the wound healing due to the easy chitosan degradation by tissue enzymes, achieving the regeneration the skin tissue of the wound area [92]. In the same way, other authors synthesized a chitosan/gelatin composite which showed good mechanical properties, to be used as artificial skin. This composite does not elicit any adverse inflammatory reactions as a consequence of its biodegradability and biocompatibility [93].

Commercially available suture materials are composed by materials as catgut, chromic catgut, polyglycolic acid and polylactic acid. The main drawback of these materials is related to their low degradation capacity. Considering that chitosan displays a higher biodegradability, chitosan based-composites are sustainable materials for use in suturing wounds.

### 3.5. Drug Delivery

The development of drugs in the clinical field is a great challenge due to the fact most drugs do not achieve desired clinical effects as a result of their inability to reach the target site of action. A high proportion of the dosed drug is disseminated over organs and tissues, which are not involved in the pathological processes, leading to severe effects.

Chitosan can also be employed as potential excipient to a sustained release for oral drugs in the form of granules or beads due to its abundant availability, inherent pharmacological properties and other biological properties (biodegradability, biocompatibility, non-toxic profile or low-immunogenicity) without side effects in the human body (Table 6). Chitosan can also be a suitable matrix in different forms (beads, films, microcapsules, coated, tables). Membranes or films can be prepared with different hydrophilic behavior by the formation of mixtures or semi-interpenetrated and interpenetrated networks of chitosan with highly hydrophilic polymers, such as polyvinyl alcohol, polyvinyl pyrrolidone or gelatin, which have controlled swelling. It has been reported that glutaraldehyde-crosslinked chitosan-gelation is pH-sensitive to drug delivery since this gel can swells and de-swells in a wider range of pH [94]. Intelligent drug delivery system can release them in reaction after some change in environmental conditions (temperature, pH, electric field, light and some chemicals). This drug delivery is administrated by the diffusion coefficient and relaxation time, which is highly dependent on the pH and the drug solubility [95].

Several chitosan-based systems have been used for these applications. Among them, chitosan/polyethyleneglycol/alginate microspheres have been described as suitable material for the delivery of compounds with low molecular weight such as heparin, which displays anti-thrombotic properties [96]. In the same way, chitosan nanoparticles have been used for the nasal administration of vaccines and drugs due to it favors the penetration of the active molecules through the nasal barrier [97]. As was previously indicated, it is possible to generate chitosan scaffolds to obtain three-dimensional supports, so these chitosan-based composites are appropriate candidates to be used as drug reservoirs. In this sense, it has been reported that chitosan-based composites can be non-viral gene delivery systems owing to the fact chitosan (positively charged) can be complexed with the DNA plasmid genes or antigens (negatively charged) [98].

Chitosan also displays great potential in cancer treatment due to its use in the design of suitable anticancer drug delivery systems as well as tracking the path of the drug carrier though a bio-friendly heavy metal free quantum dot [99]. As an example, the quantum dot formed by folic acid/carboxymethyl chitosan/Zn_x_Mn_1-x_S is used for the targeting controlled release of drugs as well as the obtaining cancer cell images [100]. In the same way, it has been reported in the literature that chitosan can activate caspase-3 protein causing apoptotic death of bladder tumor cells [101]. Some experiments with animals have demonstrated that the use of chitosan can inhibit the advance of metastatic breast cancer as well as force macrophages to mature into cytotoxic macrophages [102], which favors the suppression the growth of tumors [103]. In the same way, chitosan can favor the necrotic death of liver cancer cells due to neutralization of the cell surface charge [104]. Moreover, chitosan inhibits Enrlich ascites tumor growth due to the decrease of the glucose uptake and ATP live, diminishing the glycolysis, in tumor cells [105].

### 3.6. Artificial Kidney Membrane

Commercially both cuprophan and cellulose are used as semipermeable artificial kidneys due to their mechanical strength and good permeability. In order to improve the dialysis rates for medium and large size molecules, many polymeric membranes have been designed and developed. Considering these premises, chitosan has shown to be a promising artificial kidney membrane due to it displays an appropriate permeability to urea and creatinine as well as impermeability to serum proteins [106]. Taking into account that chitosan has widely used as resistant film, several chitosan membranes have been proposed for reverse osmosis, metal ion uptake, ion exchange, diffusion of dyes and separation of several binary systems [107]. In fact, the properties of these membranes can be optimized by the addition of some water-soluble polymers or by the grafting of copolymers that can improve the dialysis properties. Thus, it has been reported that the mixture chitosan/polyvinyl acetate displays a great tensile strength. Several authors studied the diffusion of bovine serum albumin (BSA) in highly cross-linked chitosan/polyvinyl alcohol membranes while other authors have studied the efficiency of these polymeric membranes in transporting alkaline ions and low molecular weight molecules [108]. In turn, Hirano et al. prepared chitosan-based membranes, which displayed good properties in dialysis treatment [109].

### 3.7. Blood Vessel

Vascular diseases are one of the highest causes of mortality worldwide. One of main treatment for vascular diseases is vascular transplantation. Nowadays, expanded polytetrafluoroethylene (ePTFE) and polyethylene terephthalate (PET) have been the main biomaterials used for prosthetic vascular grafts [110]. These materials display however some limitations for small-diameter applications. In this sense, complexation of glycosaminoglycans with porous chitosan scaffolds inhibits the anti-coagulant activity and vascular smooth muscle cells [111]. Other authors also fabricated a chitosan/heparin scaffolds with potential applications in blood vessel tissue engineering as hemostatic agent for vascular grafts [112].

Catheters are tubes, generally long, thin and flexible, made of different materials (rubber, plastic or metal), which is used in medicine and surgery for therapeutic or diagnostic purposes. These tubes is inserted into a duct, blood vessel, organ, or cavity to explore, widen, unclog, evacuate or inject a fluid. The use of heparin/chitosan in catheters has been very promising due to its physical compatibility and good clinical performance [113]. It has been reported that heparin/chitosan cyanoborohydride surface is a very promising catheter due to its higher compatibility with surrounding fluids and tissues. In addition, the heparin/chitosan coated polymers display excellent thromboresistence properties as well as blood compatibility for period as long as four days [114].

### 3.8. Ophthalmology

Chitosan has also interesting applications in the field of ophthalmology due to its mechanical stability, optical clarity, gas permeability-partially towards oxygen, immunologically compatibility, wettability, tear strength, tensile strength, elongation capacity and biodegradability, making this biopolymer a potential material to be used for the perfect contact lens [115].

### 3.9. Cosmetics

Chitosan is the only natural cationic polymer that turns viscous on being neutralized with acid. This fact together with the fungicidal and fungi static properties favor its use in creams, lotions, permanent waving lotions and nail lacquers [116].

### 3.10. Agricultural Applications

The use of both chitin and chitosan in agriculture is focused on four directions:

- Plant protection against diseases and plagues (pre- and post-harvest).
- Support of beneficial microorganism-plant symbiotic relationships.
- Enhancing biological control and antagonist microorganism action.
- Plant growth development and regulation.

Chitosan has shown an interesting fungicidal activity against many phytopathogenic fungi as well as antiviral and antibacterial activity. In this sense, the antimicrobial properties of chitosan and its outstanding film-creating aptitude have been exploited in the post-harvest preservation of fruits and vegetables, generating antimicrobial protection and enhancing the shelf life [117].

The presence of chitosan on soil favors symbiotic interactions between plant and microorganisms, as takes place for example in micorrizas. They also improve the action of plague-controlling biological organisms such as *Tricoderma* sp. and *Bacilus* sp. and are suitable for the encapsulation biocides, improving the efficiency in pathogenic plagues. Chitosan can also improve the metabolism of fruit or plant, which enhances germination and higher crop yields [118].

### 3.11. Food and Nutrition Applications

*N*-Acetylglucosamine coming from human milk improves the growth of bifidobacteria, which inhibit the growth of other microorganisms and produce lactase that is necessary for milk digestion. Cows’ milk has a limited amount of *N*-acetylglucosamine so the infants fed with this milk may suffer from indigestion. Some studies have reported that the addition of a small amount of chitosan to the diet improves the digestion and the intestinal microflora [119]. Other applications of chitosan related to food and nutrition are compiled in Table 7 [120].

### 3.12. Antioxidant and Antimicrobial Properties

**Figure 11:**
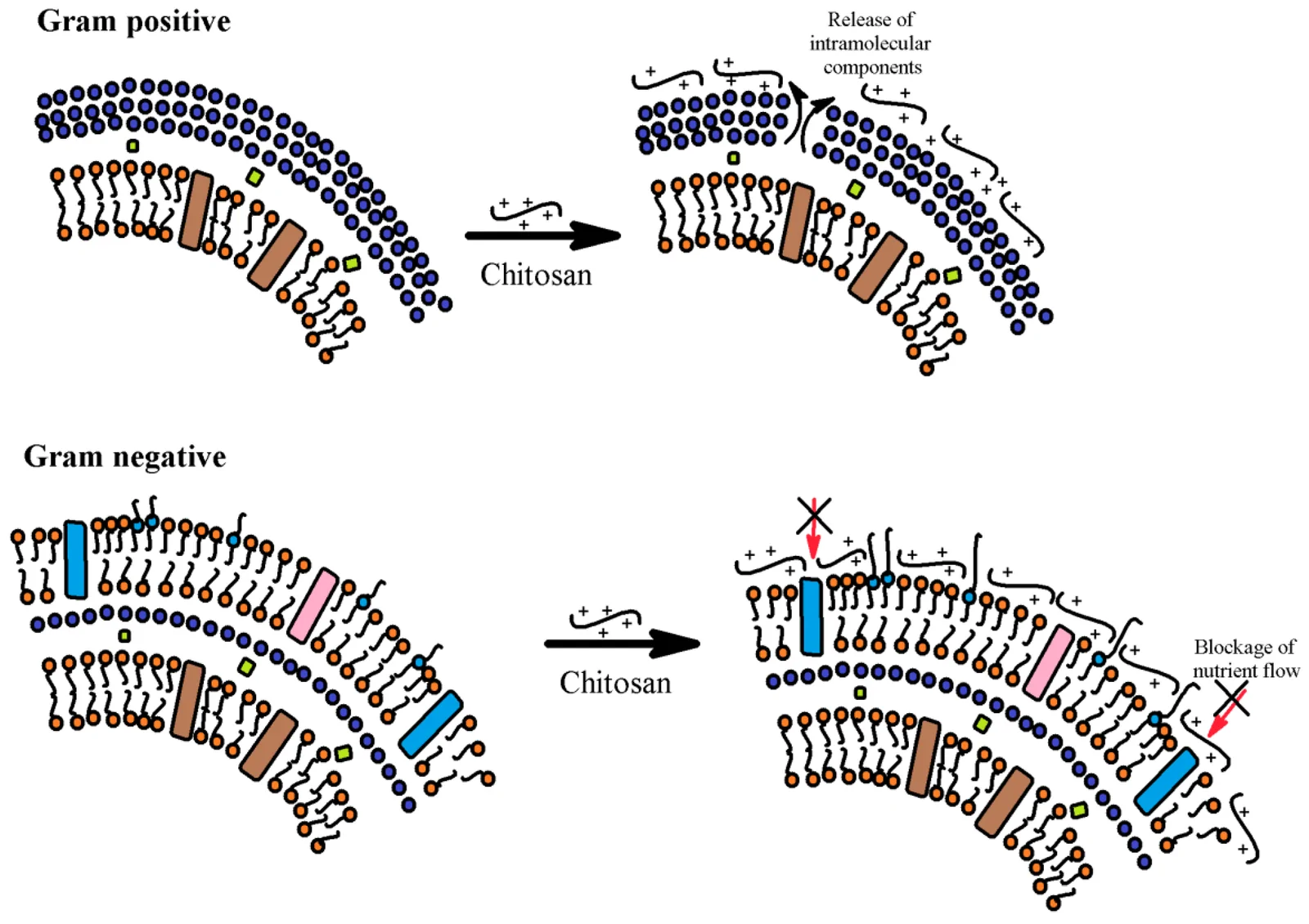
Action modes of chitosan on Gram positive and Gram negative bacteria. Structural composition of the outer envelope of Gram positive and Gram negative bacteria and effect of chitosan binding to the outer envelope of Gram positive and Gram negative bacteria.

Chitosan is a polysaccharide with antimicrobial properties. Chitosan and its derivatives exhibit differential activity towards Gram positive and Gram negative bacteria, as is evident in most studies (Figure 11) [26,121].

In Gram positive bacteria, the cell wall is made up of a thick peptidoglycan layer where negatively charged teichoic acids are covalently linked to *N*-acetylmuramic acid, while lipolyteichoic acids form covalent bonds with the cytoplasmic membrane. These teichoic acids perform functions such as providing strength to the cell wall and arranging uniform high density charges in the cell wall, thereby affecting the passage of ions across the outer surface layers [122].

In the case of Gram negative bacteria, a thin peptidoglycan layer above the cytoplasmic membrane is further covered by an additional outer envelope called the outer membrane (OM). Lipoprotein and lipopolysaccharide (LPS) are the principal components of the OM and therefore the hydrophilic O-specific side chains present in the LPS help in identifying bacteria. Hydrophobic compounds and macromolecules are usually not active towards Gram negative bacteria, and in order to interact with the Gram negative bacteria it is therefore essential to overcome the outer membrane barrier. The mode of antibacterial action of chitosan is presumably due to interactions with the bacterial surface (either cell wall or outer membrane), and to explain this mechanism [122].

This interaction modifies their barrier properties thereby preventing the entry of nutrients or causing the leakage of intracellular contents. As chitosan has the ability to form films, this biopolymer can be used to produce food packaging as a potential food preservative [123]. However, the low solubility of the chitosan film limits its applicability.

Chitosan can improve its solubility and its antimicrobial properties by the functionalization with several functional groups such as quaternary ammoniumyl, carboxyalkyl, guanidinyl, hydroxyalkyl, thiol-containing groups or hydrophobic groups (alkyl chains or benzyl rings) [124,125,126].

In the following Table 8, several antimicrobial applications are indicated.

### 3.13. Adsorption of Pigments, Dyes and Metals

As chitosan displays a polycationic structure, this biopolymer has been used as a flocculating agent but also as a chelating agent and to trap metals. Both chitin and chitosan are highly used in the treatment of wastewater. Already in 2000, chitosan was used for the removal of color in effluents coming from dye-houses [147]. In this sense, it has been reported that chitosan can agglomerate anionic wastes in solution to generate precipitates in such a way it can be used as flocculent for recycling of food processing waste [147]. Some of adsorption techniques using chitosan composites, usually with clays or polymers, have been developed to adsorb dyes as an alternative to conventional wastewater processes [148]. This interaction with the pigments and/or dyes takes place through electrostatic interaction so the pH is a key parameter in the adsorption capacity. The list of chitosan-based composites that have been tested to remove dyes in wastewater is compiled in Table 9.

In the same way, chitosan has demonstrated to be an efficient biopolymer to remove oil droplets from water. In addition, the adsorption capacity of chitosan can be improved by grafting reactions or by the formation of nanocomposites. As example, chitosan is very effective in the removal of petroleum products from wastewater by chemical interaction with the chitosan molecules [156].

As chitosan can generate resistant films in acid pH, Yang et al. synthesized reverse osmosis membranes. The obtained membrane has a NaCl rejection of 78.8%, using a flux rate of 1.67 × 10^3^ gm^3^ cm^−2^ s^−1^, a pressure of 680 psi and a salt solution of 0.2% [157].

One of the main applications of chitosan is related with the intrinsic ability of chitosan molecules to interact with transition metals through the free electron pairs that nitrogen of the amino group presents. In this sense, chitosan has been combined with clay minerals, some polymers (poly-urethane, polyvinyl alcohol) or cellulose to be employed in the metal adsorption, being highly efficient and easily regenerable [158,159,160]. In Table 10, data on the metal adsorption capacity of several chitosan-based composites are compiled.

### 3.14. Pervaporation

**Figure 12:**
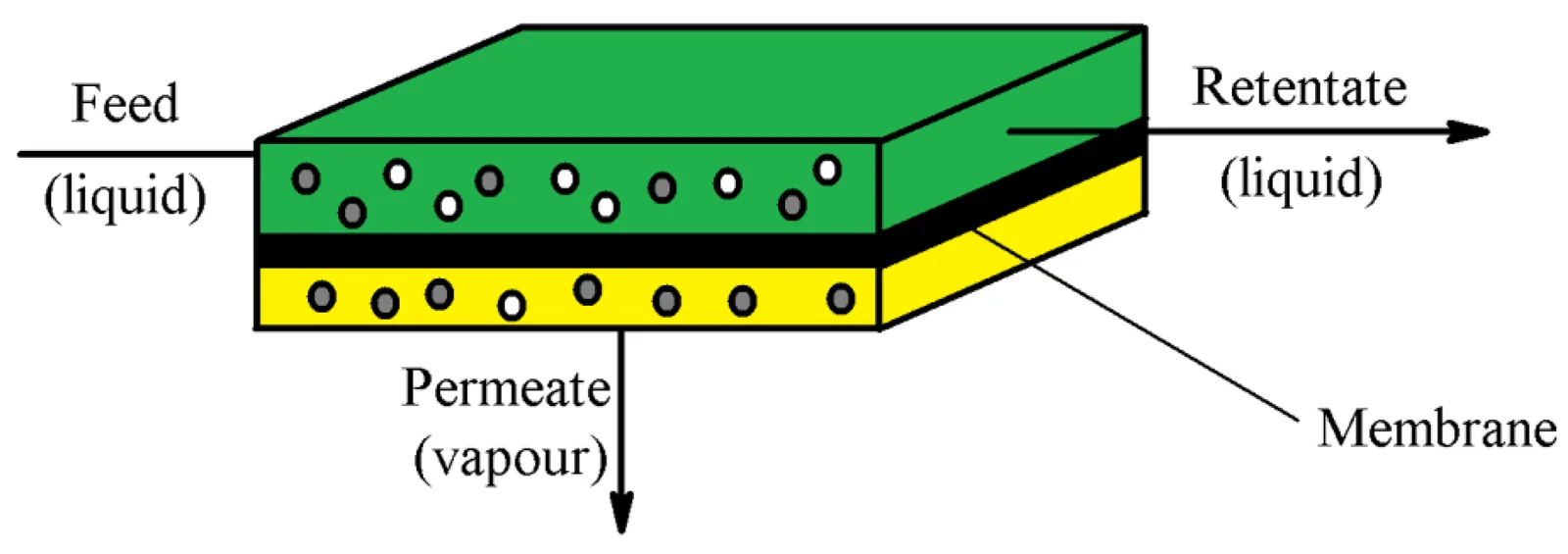
General scheme of a pervaporation.

Pervaporation is a separation method where a liquid is transported through a non-porous liophilic membrane (Figure 12). This membrane is responsible for removing some components in their vapor state into a vacuum or inert carrier gas [181]. An efficient pervaporation membrane should be characterized by a good mechanical durability, chemical resistance, high selectivity and high permeate rates [181].

Taking into account these considerations, chitosan is a hydrophilic material under acid conditions, which can react, through its hydroxyl and amine groups, with several functional groups, such as epoxy groups. The hydrophilic groups can play a key role in the preferential H_2_O sorption and diffusion through the chitosan membrane. Thus, chitosan has been proven to have good film forming properties, high water permeability and chemical resistance [182].

Many studies have reported that the chitosan membranes were highly water permselectivity or the pervaporation of aqueous alcohol solutions, as is compiled in Table 11.

### 3.15. Catalytic Applications

Chitosan can be used for both homogeneous and heterogeneous catalysis. In recent years, the scientific community is developing environmentally benign and sustainable catalysts. In this sense, the use of natural catalysts such as biopolymers is an excellent alternative to develop for the synthesis of sustainable catalysts.

**Figure 13:**
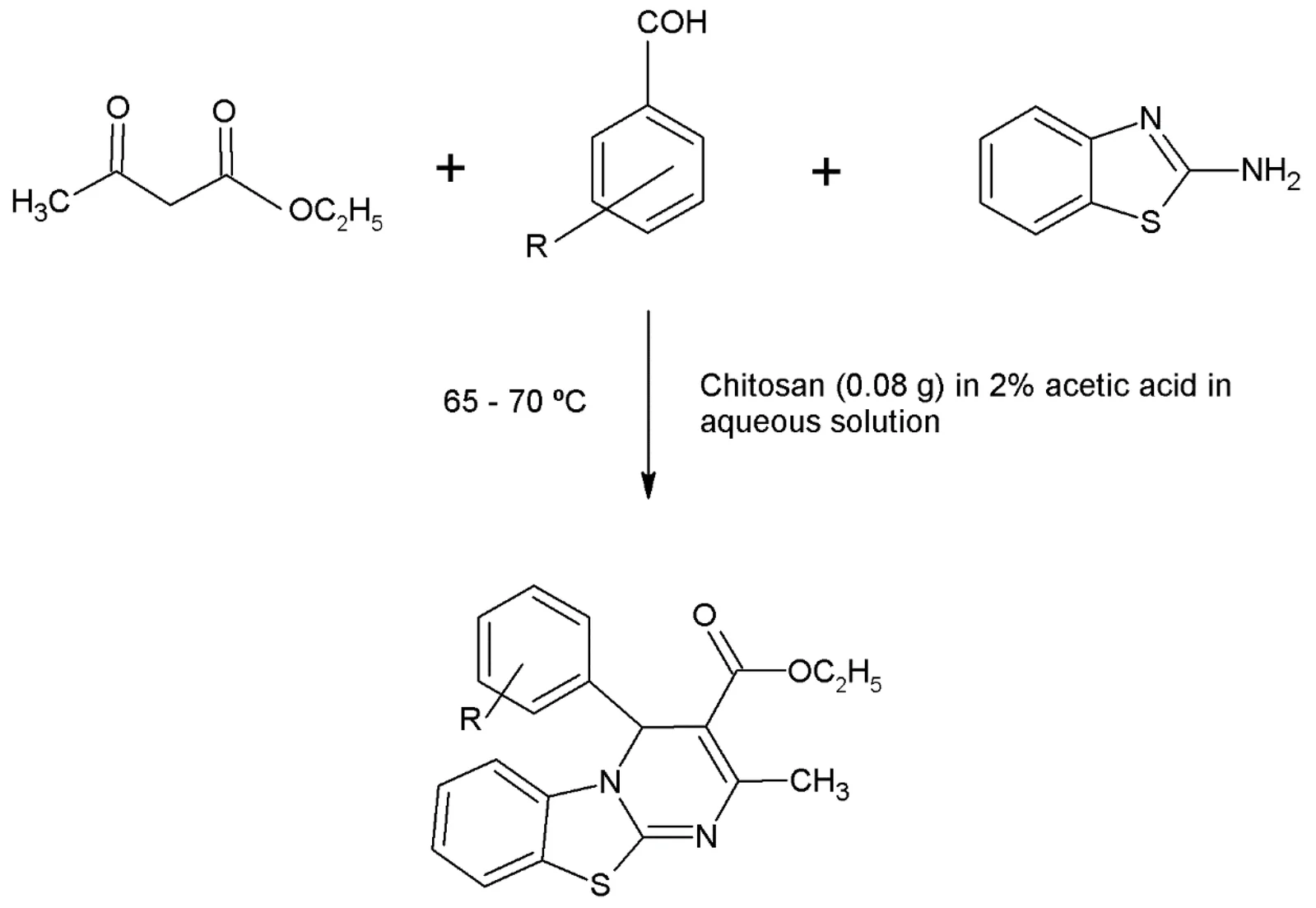
Preparation of 4*H*-pyrimido [2,1-b] benzothiazole derivatives.

Taking into account that chitosan is soluble in acetic acid solutions (minimum 1%), chitosan can act as homogeneous catalyst in the synthesis of nitrogen heterocyclic derivatives by one-pot three-component reaction of substituted aromatic aldehydes, dicarbonyl compounds and 2-aminobenzothiazole/3-amino-1,2,4-triazole/urea/thiourea in aqueous medium at 60–65 °C (Figure 13) [220]. These authors reported that free amino groups in chitosan distributed on the surface of chitosan activate the carbonyl group of benzaldehyde through nucleophilic attack to produce the corresponding intermediate [220].

**Figure 14:**
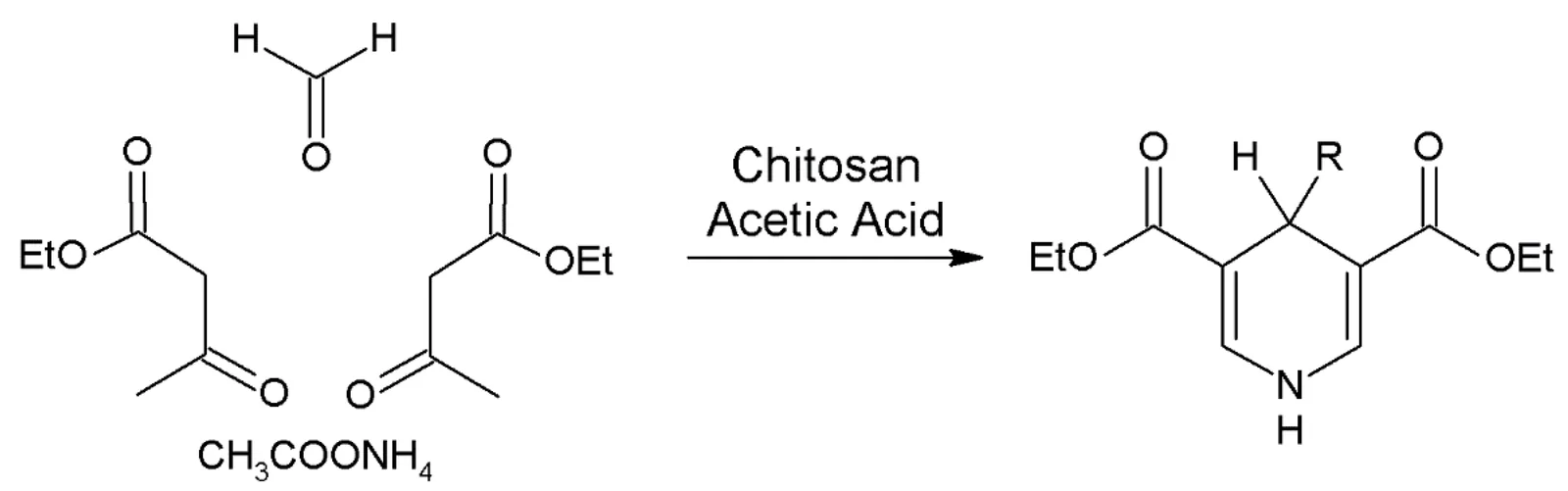
Synthesis of 1,4-dihydropyridines from the Hantzsch reaction.

Other authors have functionalized chitosan with sulphonic groups with chlorosulfonic acid obtaining a biodegradable and biocompatible acid catalyst which has been used in the condensation reaction of aldehydes, ethyl acetoacetate and ammonium acetate through the Hantzsch reaction, obtaining 1,4-dihydropyridines (Figure 14) [221].

Chitosan-based preparations loaded with metal ions and complexes as well as metal nanoparticles can be successfully used to induce different reactions. This fact favors the dispersion of the active sites (transition metal), enhancing the amount of available and increasing the availability of active sites. On the other hand, as the solubility of chitosan in water is dependent on pH conditions, these catalysts can be transformed into homogeneous in acid conditions and then it can become heterogeneous after the reaction with an increase in pH (pH > 6) to recover the catalyst. The main drawback of chitosan-based catalysts may be related to their lower thermochemical stability compared to oxides such as silica, alumina, zeolites or carbons, among others, and their low surface area. Nowadays, there are several methodologies to synthesize three-dimensional chitosan scaffolds to increase the surface area and pore volume (Figure 10).

These organocatalysts are efficient in a wide range of transformations such as organic synthesis, electrochemical reactions and decontamination reactions [222]. The presence of functional groups of the backbone affords a strong interaction with metallic species and nanoparticles. The possibility to obtain a varied chitosan-hybrid preparation further widens the options for catalyst synthesis and their use.

As was previously indicated, chitosan is a biopolymer that can be transformed into membranes, flakes, disks, hollow spheres or fibers. In addition, it can be treated to form particles of appropriate size, to develop porous structures or to increase the surface area. In the same way, chitosan can be modified, via cross-linking with different organic compounds (glutaraldehyde, glyoxal, hexamethylene diisocyanate or epichlorohydrin). This fact improves both the thermal and mechanical stabilities for chitosan [223]. In the next subsection, the chitosan-based metal catalysts involved in organic transformations are shown.

#### 3.15.1. Carbon-Carbon Coupling Reactions

Carbon-carbon coupling reactions enable the construction of organic molecules with a wide range of applications. Most of catalysts studied in the literature are based on palladium although works by using other metals such as nickel, cobalt, copper or gold have also been reported.

**Figure 15:**
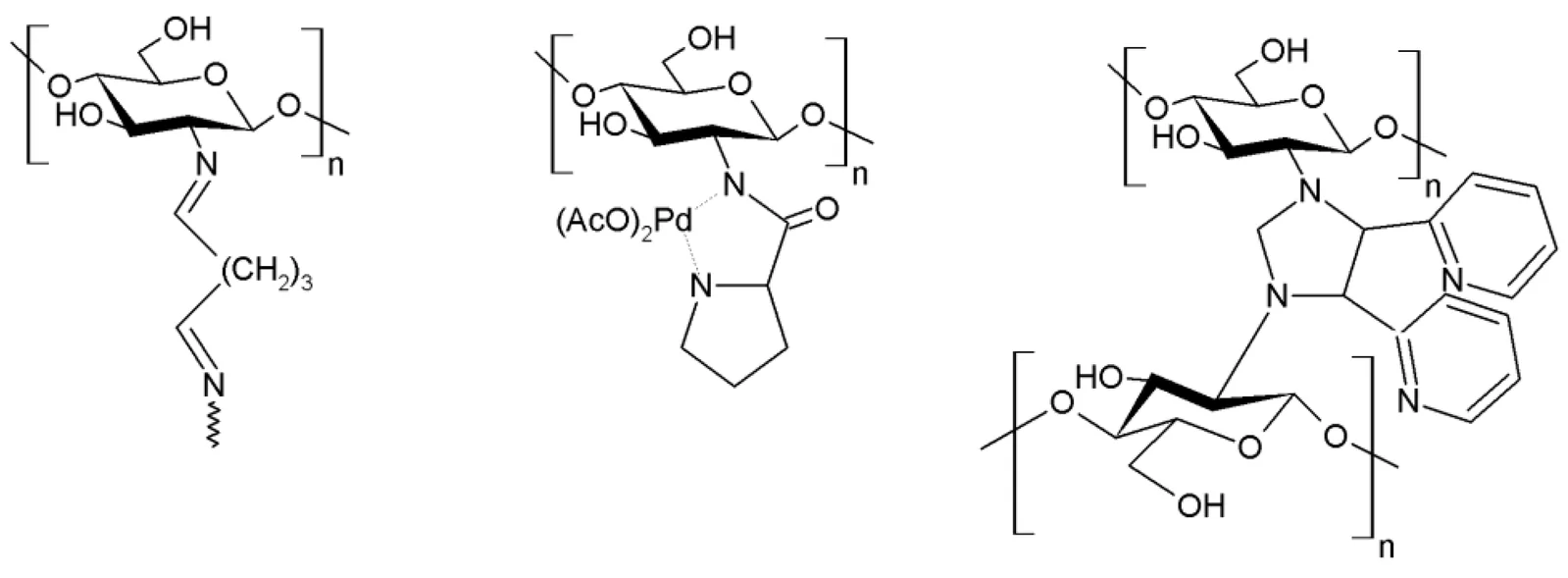
Some examples of chitosan-based catalysts loaded with Pd^2+^.

*O*-Carboxymethyl chitosan has been used as support to graft PdCl_2_ or Ni(OAc)_2_, obtaining high yields in the Heck coupling [224]. In another research, chitosan was cross-linked with glutaraldehyde to the immobilization of Pd^2+^-species in its structure, reaching yields very close to 100% (Figure 15) [225]. Other authors also synthesized proline-loaded chitosan beads and gelatin chitosan beads by cross-linking with similar results in couplings reactions of iodo- and bromoarenes, attaining conversion values between 68–100% [226,227].

**Figure 16:**
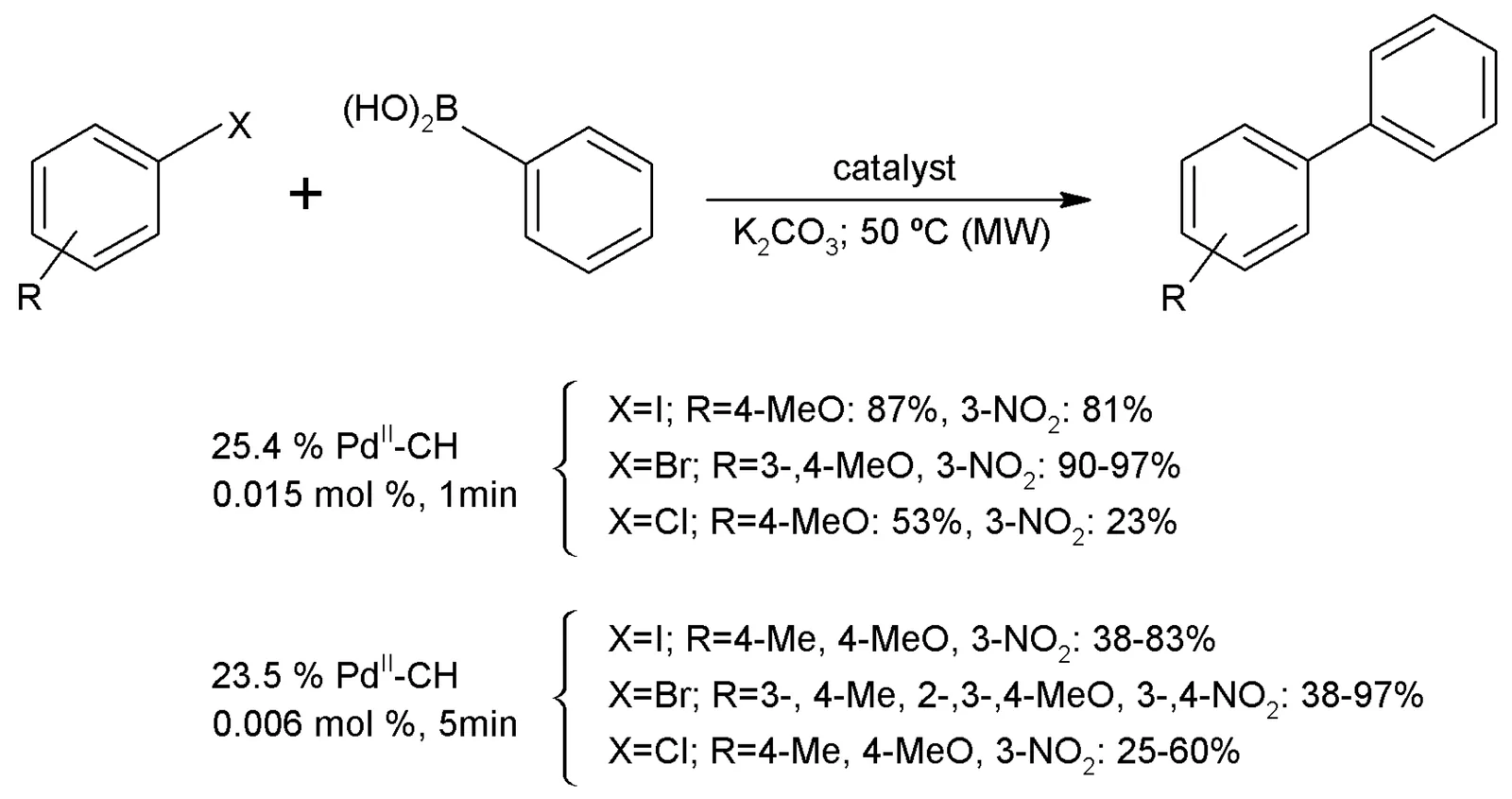
Suzuki couplings using catalysts modified with 6-carboxymethylchitosan.

In the same way, 6-caboxymethylchitosan was also modified with various Schiff bases loaded with Pd^2+^, obtaining catalysts with high activity in the Suzuki coupling reaction, although these reactions required long reaction times [228] (Figure 16).

In the same way, Zeng et al. prepared porous chitosan microspheres by cross-linking and subsequent CuI deposition, obtaining catalysts with high activity in the Heck reaction of iodobenzenes, although they required severe catalytic conditions (140 °C and 10 h) in such a way that they detected a partial leaching after the third cycle [229].

**Figure 17:**
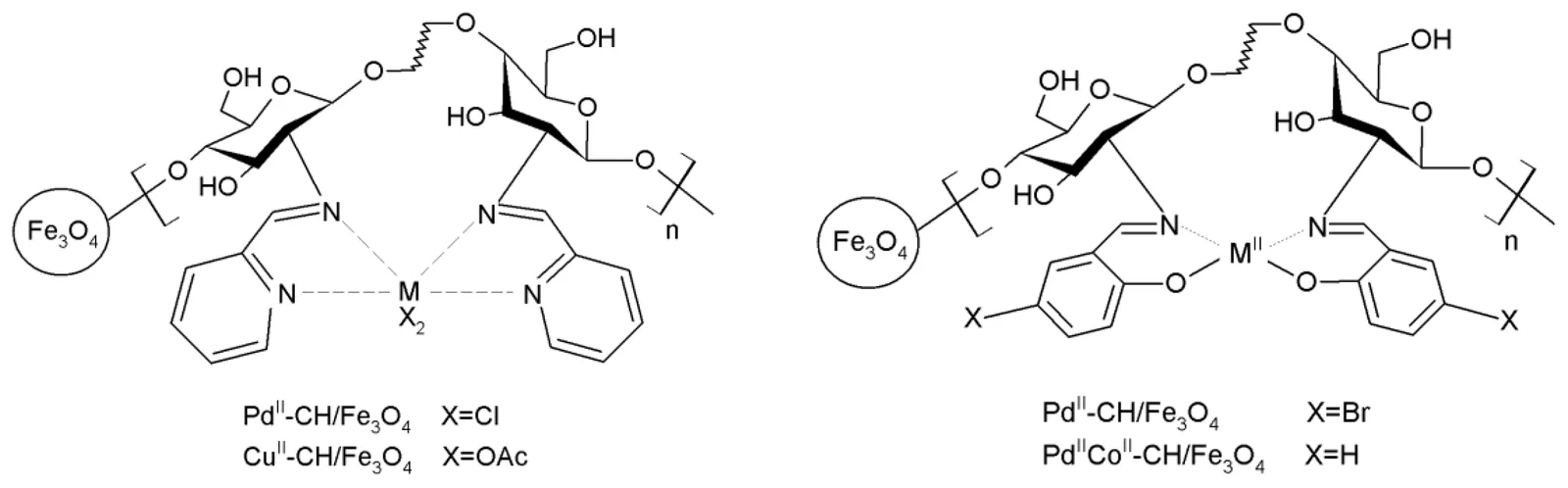
Magnetic chitosan-based metal catalysts with high activity in the Heck coupling reaction.

In order to recover these chitosan-based metal catalysts, Raffiee and Hosseini [230] carried out a reflux chitosan with cyanuric chloride in toluene and then, the product was treated with *N*-methylimidazones i-Pr_2_EtN and with magnetic Fe_3_O_4_ particles in acetic acid to recover the catalyst magnetically (Figure 17). These catalysts provided high yields (90–98%) in the Suzuki coupling of iodo- and bromobenzenes. In the same way, other authors also synthesized other magnetical chitosan-based metal catalysts (Pd^2+^, Cu^2+^ or Pd^2+^-Co^2+^) with high activity in the Heck coupling reaction [231]. Other authors have employed silica nanospheres to disperse the active phase involved in the coupling reaction. Jadhav et al. dispersed silica in an acid solution of chitosan and then induced the chitosan deposition. In the next step, the solid was treated with Pd(OAc)_2_ to obtain a mixture of Pd^2+/0^-species [232]. The obtained material exhibited a good behavior in the Heck coupling reaction with a yield of 81–92%.

**Figure 18:**
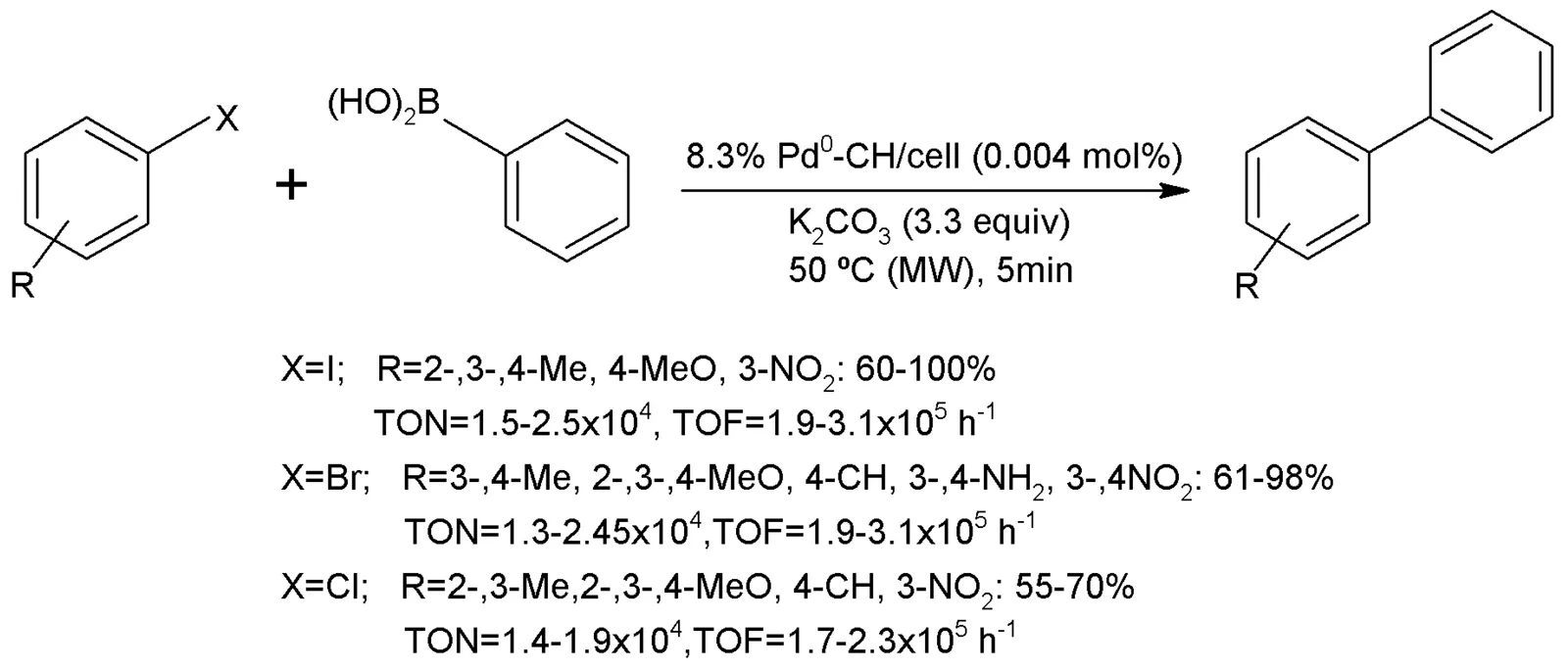
Suzuki reaction with Pd^0^-chitosan based catalysts.

Clay minerals were also used to disperse the chitosan-based catalyst. Zeng et al. synthesized a composite catalyst by the mixing of chitosan, montmorillonite and Na_2_PdCl_4_, obtaining good results in the Heck coupling of iodo- and bromo arenes, although they observed significant losses of Pd-species after each cycle [233]. Most metal-based catalysts show improved catalytic behavior in the metallic state so the complexed metal ions should be treated with a reductant such as alcohols, hydrazine or NaBH_4_ (Figure 18) [234,235].

**Figure 19:**
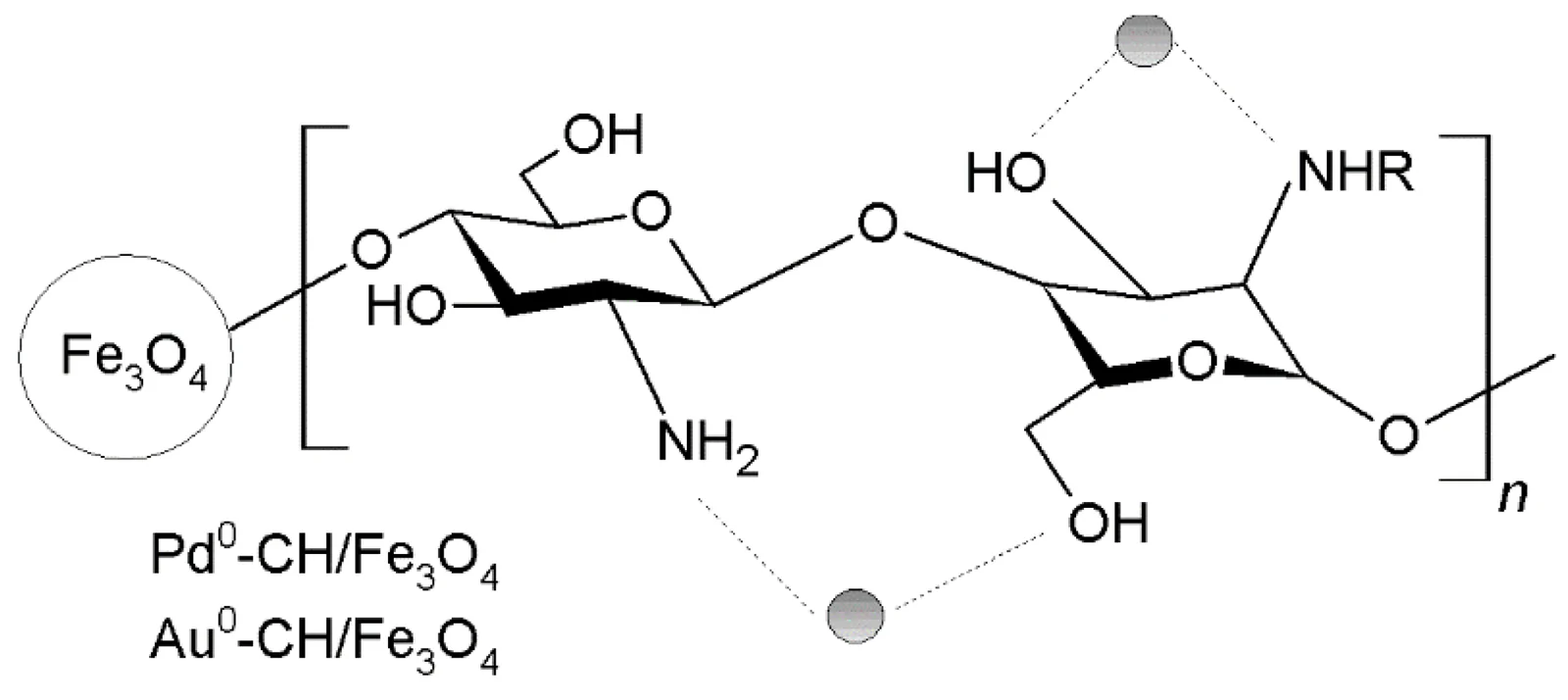
Magnetic chitosan based catalysts loaded with Pd^0^ or Au^0^ used in the oxidative homocoupling of phenylboronic acid.

**Figure 20:**
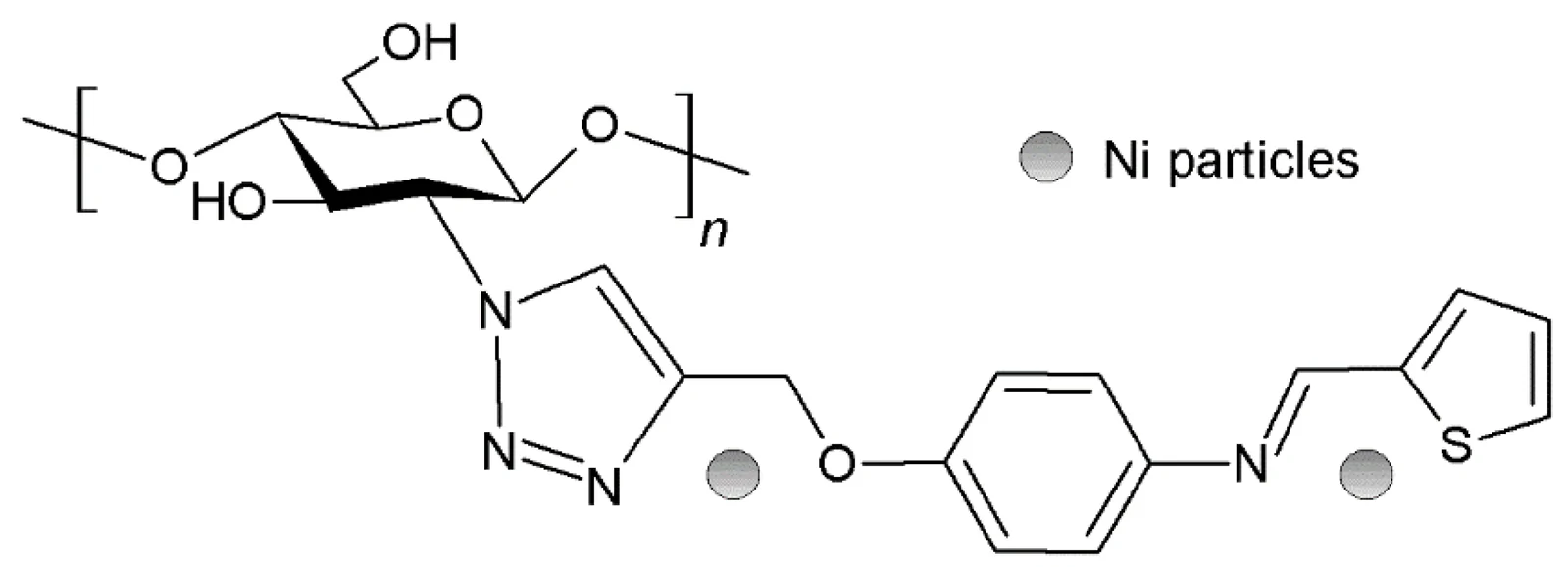
Chitosan-based catalysts loaded with Ni^0^ used in C-C. coupling reactions.

Similarly to Pd^0^, other transition metals have been employed to synthesize chitosan-based catalysts. Thus, both Au and Au-Pd bimetallic catalyst were synthesized by co-reducing of HAuCl_4_ and H_2_PdCl_4_ with NaBH_4_ in an acidic chitosan solution and then were used in the oxidative homocoupling of phenylboronic acid, reaching yields of 96–99% (Figure 19) [236]. Ni-nanoparticles have been rarely used in coupling chemistry. However, Hajipour and Abolfathi synthesized alkynylated imino thiophene ligand, transforming chitosan with azidation and then combining these two components via a click reaction (Figure 20) [237].

#### 3.15.2. Carbon-Nitrogen Coupling Reactions

**Figure 21:**
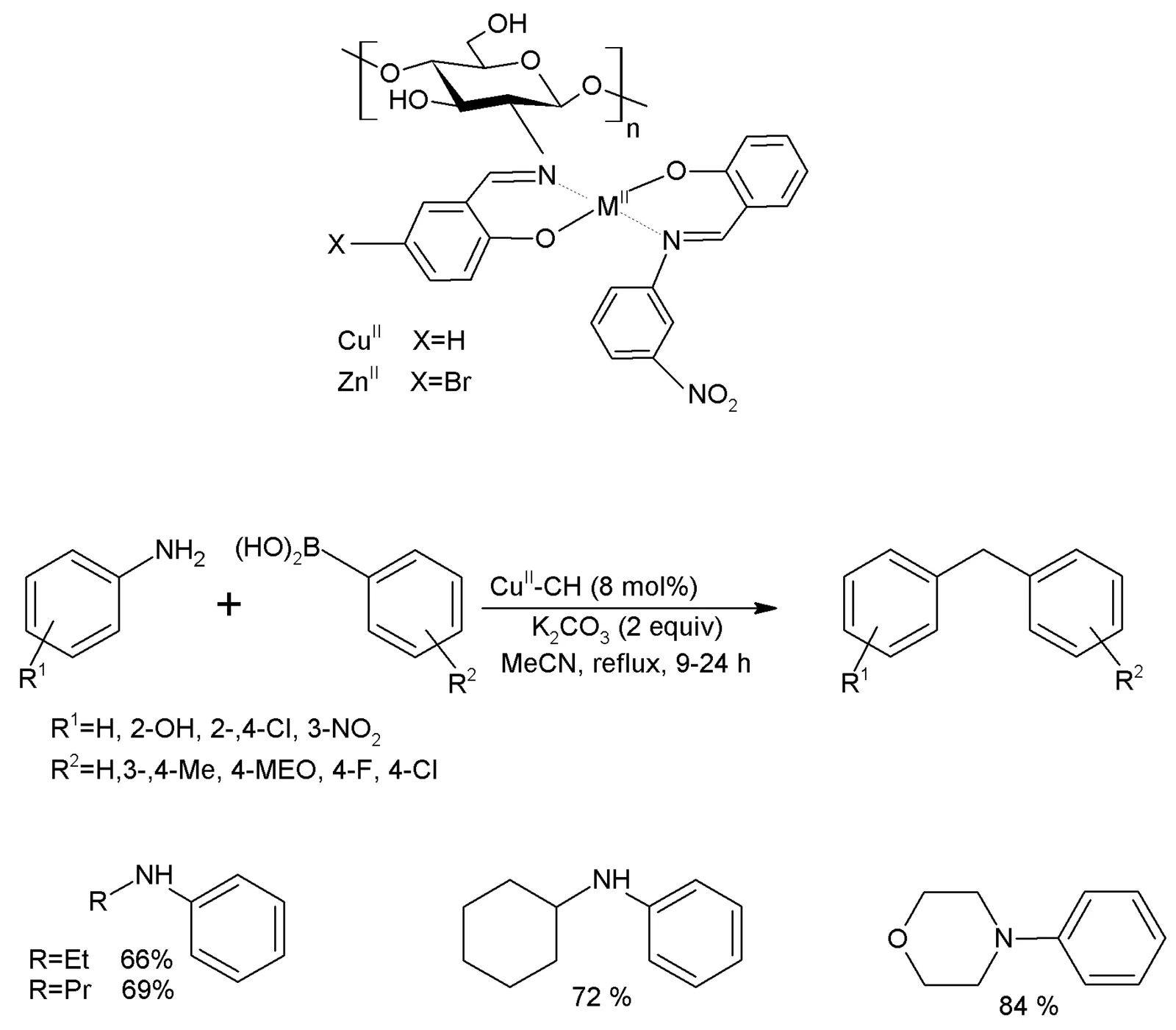
C-N coupling of amines with phenylboronic acids using chitosan-based catalysts loaded with Cu^2+^.

The most important and frequently used method to form C-N bonds via is the coupling of haloarenes. From this reaction it is possible to synthesize a wide range of amines through Buchwald-Hartwig amination. The first studies were carried out by mixing of chitosan with an ionic liquid (IL[bmin]BF_4_) and grafting with Pd(OAc)_2_ and a ligand, reaching a conversion of 98% [238]. Later, other authors reported that the incorporation of Co^2+^-species improved the yield and the stability of the catalysts in these C-N coupling reactions [239]. In another study, chitosan was functionalized with salicylaldehyde, obtaining a complex, which was stirred with 3-nitroaniline in ethanolic solutions of Cu(AcO)_2_, obtaining a catalyst with high yields in the reaction of primary amines with phenylboronic acids [240]. In the same way, Bodhak et al. treated chitosan with CuSO_4_ in aqueous solutions to form a catalyst, which was employed in the coupling of iodo- and bromo-benzene with aliphatic 1,2- and 1,3-diamines, achieving a yield of 86–94% (Figure 21) [241].

**Figure 22:**
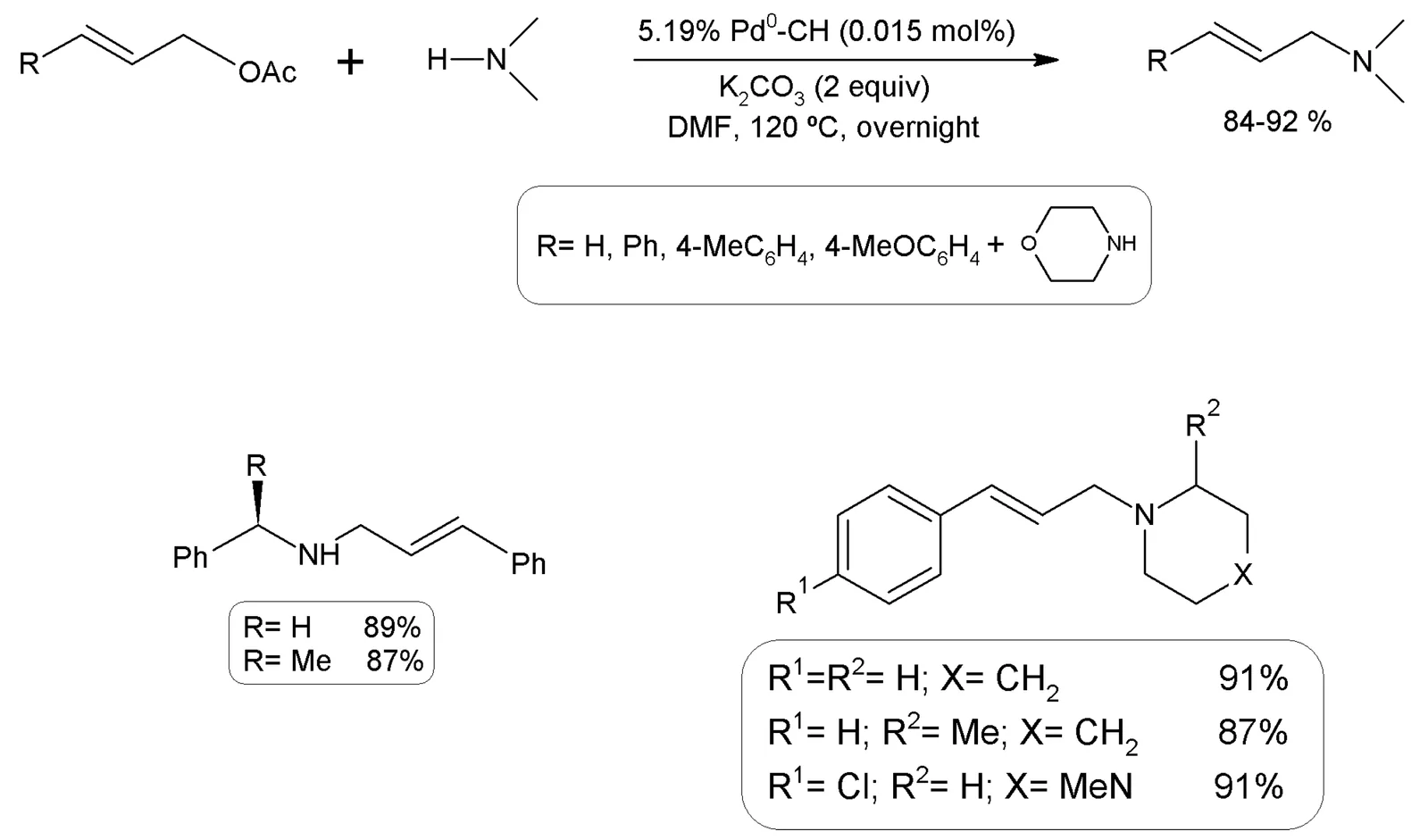
Coupling of allyl acetates with amines using chitosan-based catalysts loaded with Pd^0^.

Palladium particles generated by the reduction of PdCl_2_ with NaBH_4_ in the presence of chitosan followed by drying afforded catalysts, obtaining high yields in the coupling of allylic acetates with phenylethylamines and N-heterocycles, reaching yields of 78–86% after five cycles (Figure 22) [242].

#### 3.15.3. Carbon-Sulfur Coupling Reactions

Recently, Cu-based catalysts have been employed in the carbon-sulfur coupling reactions. Zhang et al. synthesized a catalyst from Cu(OAc)_2_ and chitosan, obtaining Cu-particles of 3–8 nm [243]. These catalysts showed a good catalytic behavior in the reaction of iodo- and bromoarenes with sodium sulfinates to produce sulfones.

**Figure 23:**
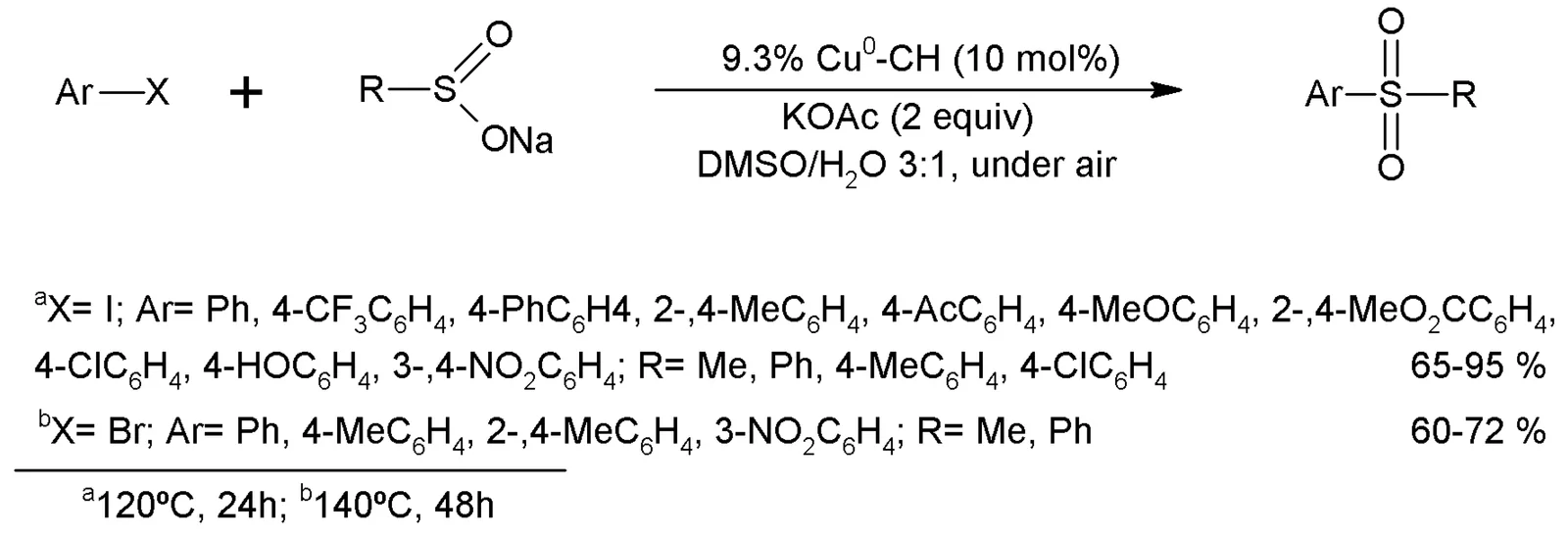
Coupling of haloarenes and sodium sulfonates using chitosan-based catalysts loaded with Cu^0^.

In the same way, García et al. prepared Cu nanoparticles of about 0.5 nm y solvothermal reduction of an acidic chitosan/Cu(NO_3_)_2_ solution. Then, the gel was treated with an alkaline solution to form microspheres. The obtained materials showed a high surface area and a good activity in the coupling of iodobenzene and thiophenol, reaching a yield of 96% (Figure 23) [244].

#### 3.15.4. Oxidation Reactions

Chitosan-based metal catalysts have also been employed in oxidation reactions. One of the biggest challenges that must be addressed is related to the stability of chitosan under oxidative conditions. This has been prolifically studied with the aim of transforming chitosan, insoluble in water, to low-molecular weight products through hydrolysis. In this sense, high degradation was achieved with H_2_O_2_ under acidic conditions (pH = 5). The first studies of oxidation with the aid of chitosan-based catalysts was reported by Pispisa applying immobilized Fe^3+^ and Cu^2+^ complexes in alkaline solutions for the oxidation of catecholamines via intramolecular electron transfer [245].

Several studies have used porphyrin-chitosan in preparation of the oxidation of cyclohexane. In all cases, several cations (Fe^3+^, Mn^3+^ and Co^2+^) were complexed in the tetraphenylprophyrin ring, obtaining the highest activity for the Fe-based catalyst. In later studies, this group immobilized Fe^3+^-tetrakis(4-carboxyphenyl)porphyrin, obtaining a material with higher porosity and higher amount of available active sites [246]. This catalyst showed better activity values in the oxidation reaction of cyclobenzene or ethylbenzene [247]. In the same way, Shaabani et al. synthesized chitosan-based cobalt catalyst by reducing the aqueous solution of chitosan mixed with CoCl_2_ with NaBH_4_, being active in the aerobic oxidation of benzylic carbon atoms to form their corresponding ketones and aldehydes with a yield of 90–95% [241].

Crucianelli et al. synthesized silica-supported preparations with chitosan bearing O-acyl, N-acyl and N-alkyl moieties by complexation with methyltrioxorhenium. Oxidation of alkenes were carried out with urea-H_2_O_2_ adduct providing high epoxide yield in ethanol at room temperature [248]. In the same way, Ru^3+^-based catalyst also showed interesting results in epoxidation reactions with NaIO_4_ [249].

Thiol-functionalized chitosan was prepared by modification with methyl acrylate and then the adduct was functionalized with ethane-1,2-thiol. The obtained product was loaded with AuCl_3_ and reduced with NaBH_4_. The obtained catalyst was very active and stable for several cycles in oxidation of cyclohexene [250].

**Figure 24:**
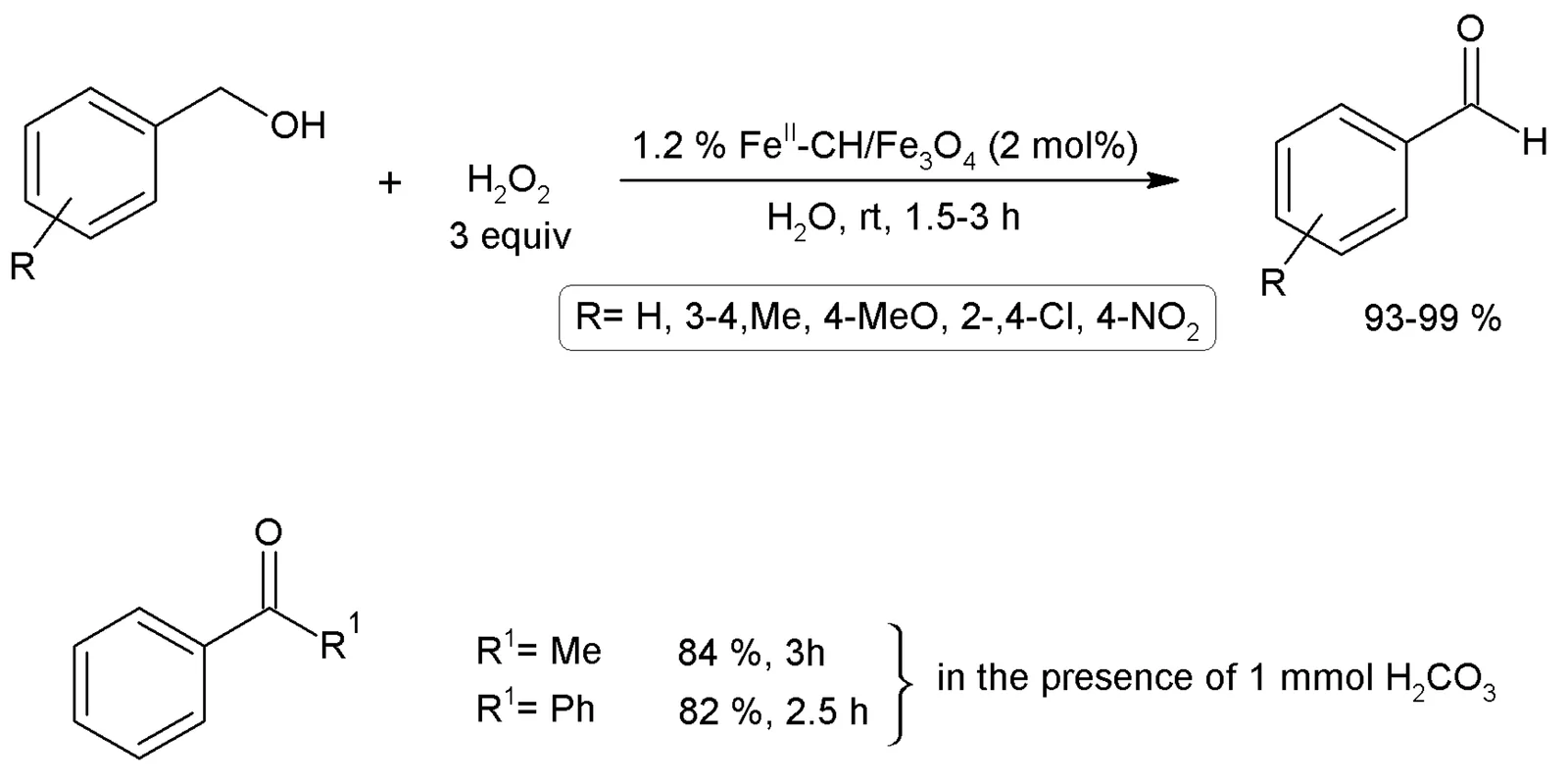
Selective oxidation of benzyl alcohols to carbonyl compounds using chitosan-based catalysts loaded with Fe^3+^.

The oxidation of alcohols by using chitosan-based metal catalysts was firstly described by the Shaabani group in the aerobic oxidation of benzylic alcohol [251]. It has been reported in the literature that Fe^3+^ are active species in the oxidation of benzyl alcohol to their respective carbonyl compounds (Figure 24). In the same way, both Au and bimetallic AuPd catalysts in the oxidation of 4-hydroxybenzyl alcohol to obtain 4-hydroxybenzaldehyde in the presence of K_2_CO_3_ [252]. On the other hand, porous chitosan/polyacrylamide interpenetrating polymer network with entrapped silver nanoparticles was active in the oxidation of 1-phenylethanol for at least 7 runs [253].

#### 3.15.5. Hydrogenation Reactions

In this section, chitosan-based metal catalysts involved in hydrogenation of C-C and C-X are summarized. RhCl_3_(H_2_O)_3_ was mixed with chitosan and then reduced with NaBH_4_ under supercritical CO_2_ conditions to afford a catalyst with high surface area [254]. This catalyst showed good catalytic activity in the hydrogenation of buta-1,3-diene and but-1-yne both in gas and liquid phases. On the other hand, chitosan solution in 3 wt.% formic acid treated with H_2_PdCl_4_ or H_2_PtCl_6_ were employed in the hydrogenation of unsaturation of palm oil [255].

**Figure 25:**
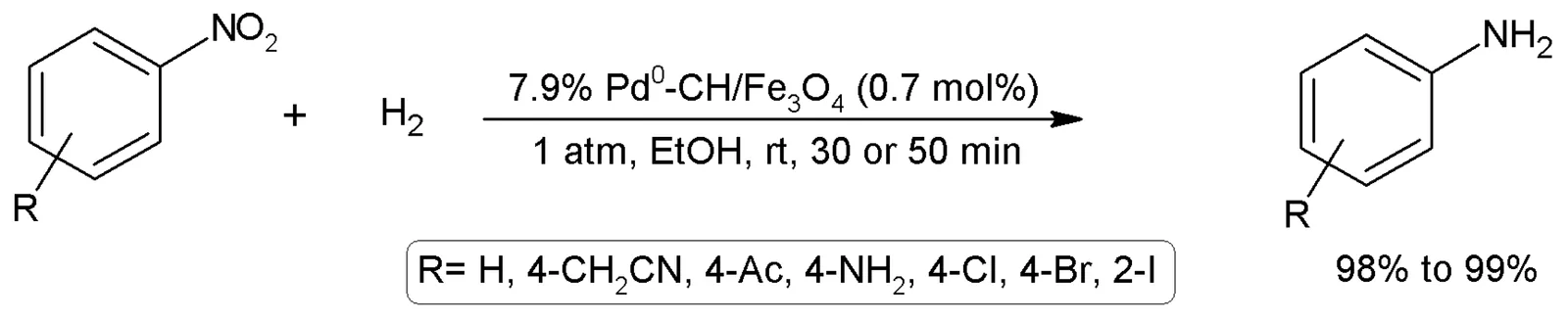
Hydrogenation of nitroarenes using chitosan-based catalysts loaded with Pd^0^.

Adlim and Bakar reported mono- and bimetallic Pd, Au and Pd-Au catalysts stabilized by chitosan and subsequently reduced with methanol or NaBH_4_ [256]. The obtained catalysts, mainly the bimetallic ones, exhibited relevant values of dispersion and activity in the hydrogenation of octa-1-ene. In the same way, other authors synthesized a magnetic chitosan-coated Fe_3_O_4_, with H_2_PdCl_4_, and then, Pd^2+^-species were reduced with NaBH_4_. This catalyst displayed a high dispersion of the Pd^0^ (Figure 25), which provided a high activity in the hydrogenation of nitrobenzenes to form the corresponding amines [257].

**Figure 26:**
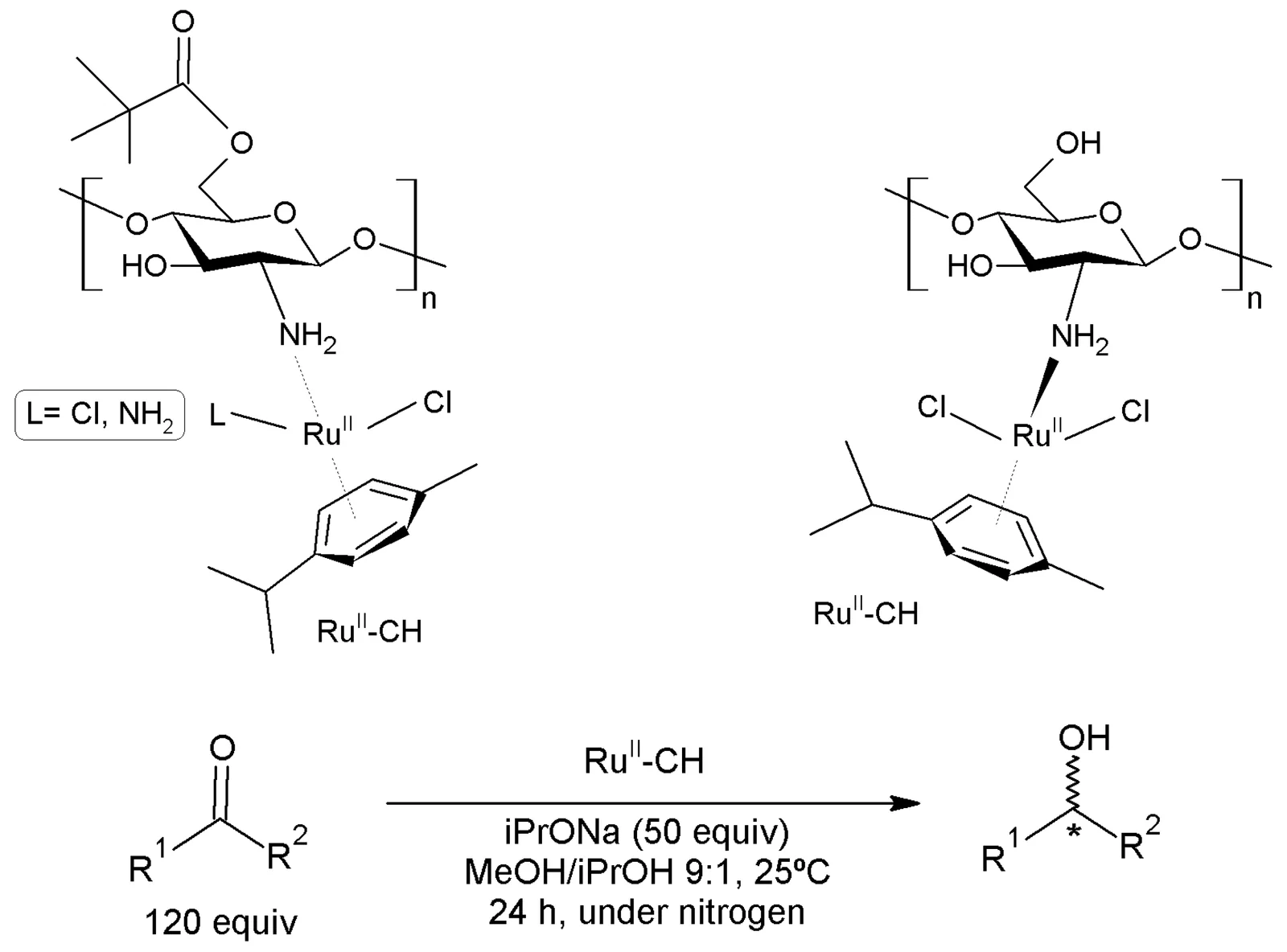
Chitosan-based catalysts loaded with Ru used in transfer hydrogenation reactions.

It has been reported in the literature that chitosan-based Ru catalysts show interesting results in transfer hydrogenation reactions. A Ru^2+^-complex was prepared by esterification of chitosan with pivaloyl chloride (Figure 26). Then, this adduct was treated with [Ru(*p*-cymene)Cl_2_]_2_ in methanol. The obtained product was tested in the asymmetric transfer hydrogenation of acetophenone [258,259]. One of the main drawbacks of this catalyst were its degradation and precipitation under basic conditions [258].

#### 3.15.6. Hydrogenolysis Reactions

Likewise, a slurry containing chitosan with PdCl_2_ adsorbed in ethanolic solution was reduced with NaBH_4_ at room temperature, being active in the transformation of 2-phenyloxirane to 2-phenyl-ethanol, which is used in the fragrance industry [260]. On the other hand, Santes et al. synthesized a multicomponent (Mo, Ni and P) chitosan-based catalyst by pore-filling impregnation, being tested in the hydrodesulfurization of dibenzothiophene to obtain high proportion of biphenyl [261].

### 3.16. Catalytic Processes to Valorize Chitosan into Valuable Products

In recent years, the scientific community is developing processes to valorize lignocellulosic biomass to obtain high-added value products. These processes are encompassed in the biorefinery concept. Considering that chitosan has a structure analogous to the sugars obtained from cellulose and hemicellulose, chitosan has great potential to obtain products with high commercial interest.

**Figure 27:**
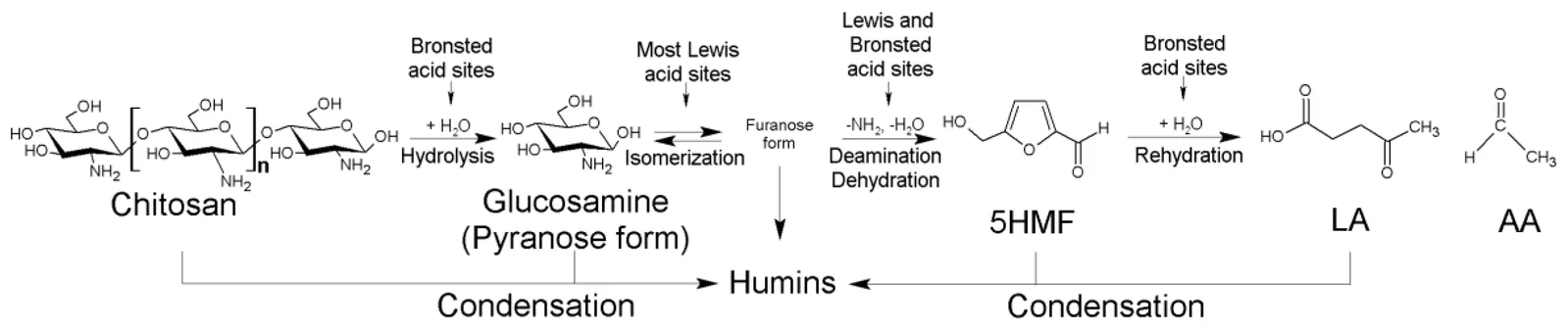
Scheme for the production of HMF and LA from chitosan.

The hydrolysis and dehydration processes of chitosan to produce 5-hydroxymethylfurfural (HMF, Figure 27) were firstly performed using mineral liquid acids, such as H_2_SO_4_ [262]. The optimization of reaction parameters led to an HMF yield of 14% after 37 min of reaction by using 2.2 wt.% of H_2_SO_4_ [263]. Ionic liquids have shown a higher activity than mineral acids, giving rise to a HMF yield of 29.5% from chitosan and 19.3% from chitin, after 5 h of reaction at 180 °C using *N*-methylimidazolium hydrogen sulfate ([Mim]HSO_4_) as catalyst [264]. Other authors have added ZnCl_2_ as a homogeneous catalyst, where Zn^2+^ ions are coordinated with -OH in C1 and -NH_2_ in C2, obtaining a HMF yield between 2.8–10.1% after 1.5 h of reaction at 120 ° C from the chitin monomers (GlcNAc) and chitosan (GlcNH_2_) [264]. Similarly, other authors evaluated several metal chlorides and boric acid as co-catalysts, obtaining the highest conversion values for AlCl_3_ and boric acid, with a maximum yield towards HMF of 26.5% from GlcNH_2_ [264]. Another catalyst with high activity is SnCl_4_-5H_2_O, which is hydrolyzed under reaction conditions to form SnO_2_, with a HMF yield of 13.2%, using chitosan with high molecular weight [265].

**Figure 28:**
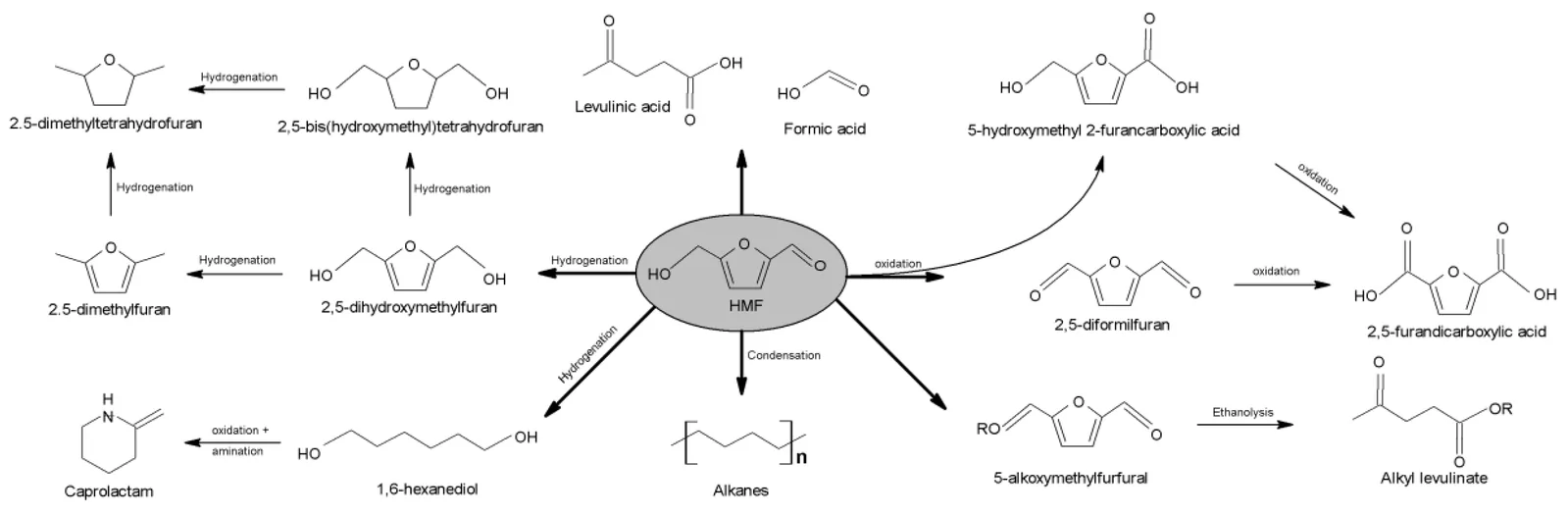
Scheme showing possible reactions of 5-hydroxymethylfurfural to obtain high value-added products.

HMF is considered one of the most versatile biological-based compounds [266], since it can be transformed into a wide range of chemicals (Figure 28) [267]. In this context, together with cellulose and hemicellulose, both chitin and chitosan are also appropriate biopolymers for the synthesis of valuable furan derivatives.

**Figure 29:**
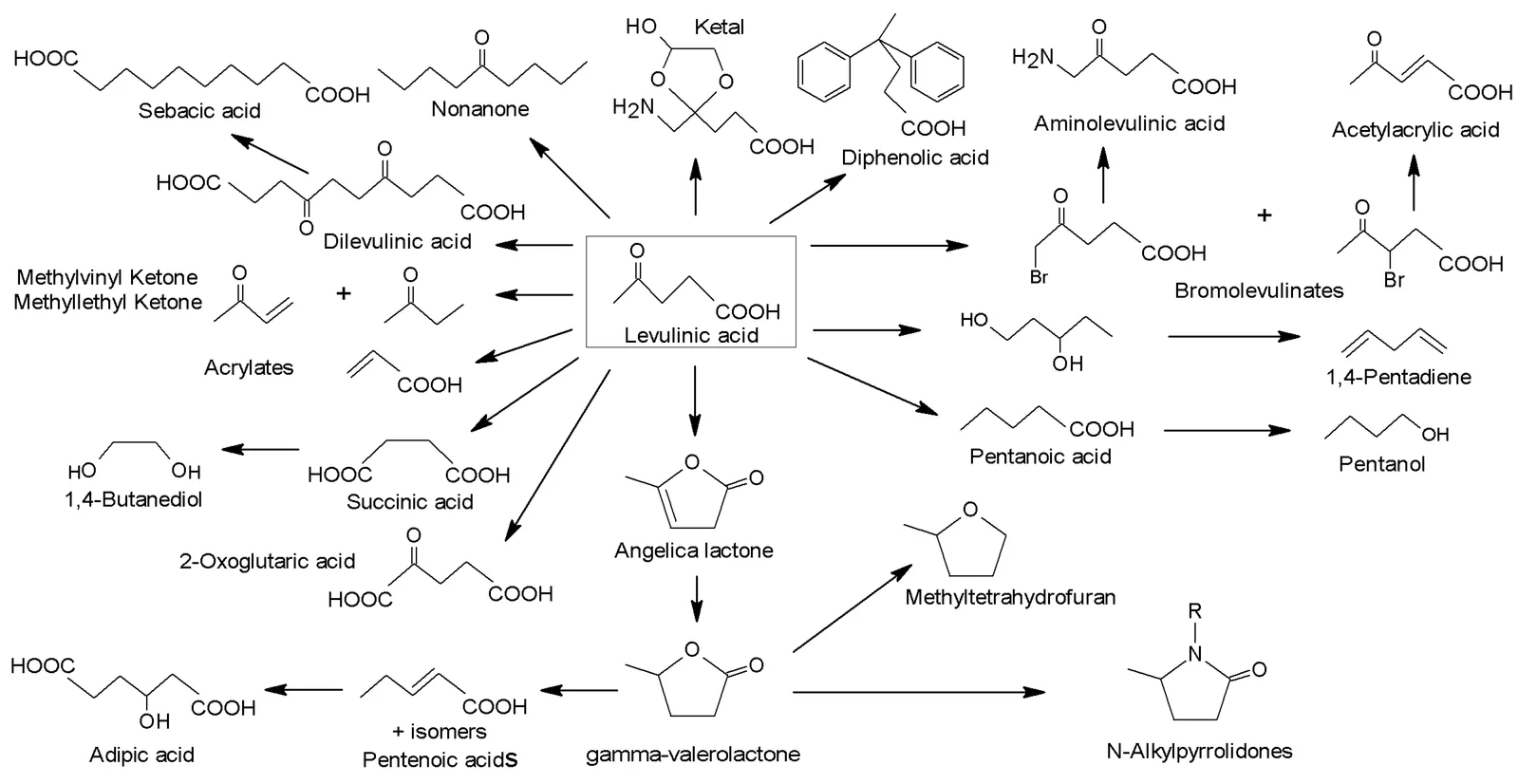
Scheme of the possible reaction of levulinic acid to obtain high value-added products.

The subsequent rehydration of HMF could also give rise to levulinic acid (LA), which is considered another useful building block molecule, since it can be converted in many different value-added products (Figure 29). In this sense, it has been reported that LA can be obtained from chitin and chitosan in the presence of H_2_SO_4_ (2 M) at 190 °C for 30 min [268]. LA was also the major product when SnCl_4_ was used as catalyst [269], achieving yields of 34.7% and 59.4% from chitosan and chitosan monomer respectively, under microwave irradiation at 220 °C. In another study, a LA yield of 39% was achieved through the hydrothermal transformation of GlcNH_2_, and 29% when chitin was hydrothermally treated in 4 wt.% H_2_SO_4_ solution at 190 °C [270].

**Figure 30:**
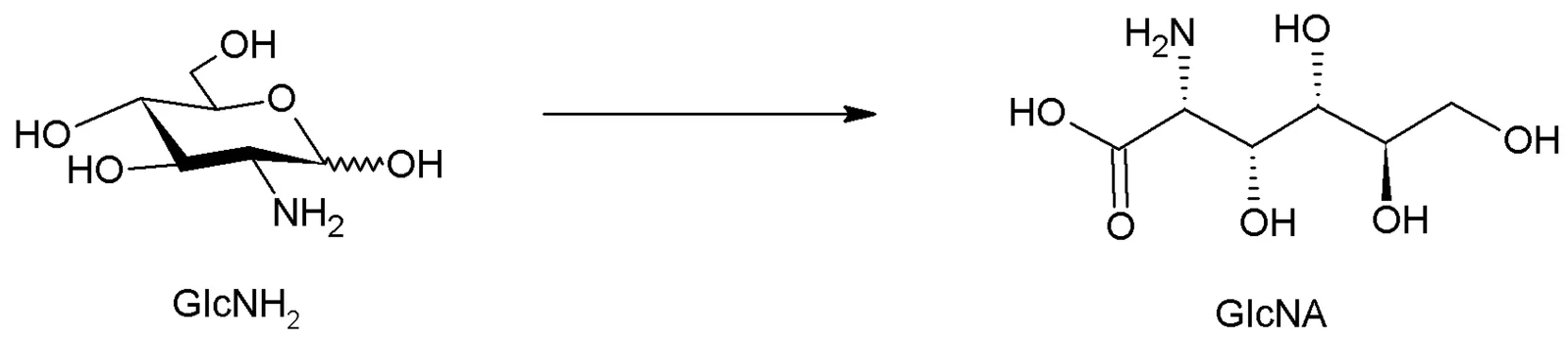
Oxidation of GlcNH_2_ to GlcNA.

Chitin and chitosan can also undergo oxidative reactions in the presence of noble metal nanoparticles, generally Au, dispersed on metal oxides, leading to 2-amino-2-deoxy-d-gluconic acid (GlcNA) (Figure 30), which is used in the asymmetric synthesis of amino acids, besides its potential in biomedical applications [271].

Traditionally, Au nanoparticles have shown to be active in the oxidation of alcohols. Thus, the oxidation of glucose to gluconic acid has been extensively reported. However, this oxidation reaction has hardly studied using an analogous monomer, such as GlcNH_2_, where one –OH group is replaced by –NH_2_. As this field is very little explored, this task has a great potential to design novel catalysts to synthesize these valuables starting products for fine chemistry.

## 4. Conclusions

Chitosan is a natural polymer with tremendous biological properties such as biocompatibility, biodegradability and anti-infective activity, among others, due its high charge density, the existence of reactive hydroxyl and amine groups or its capacity of interact with other polymeric fractions through hydrogen bonds. These interesting physicochemical properties and the capacity to functionalize the amine and hydroxyl groups for specific uses has attracted the interest of the scientific community in the last decades to study and develop interesting applications for chitosan.

In this review, it has been highlighted that chitosan and chitosan-based composites have enormous potential in medical applications such as burn treatment, artificial kidneys, blood anticoagulation and bone, tendon or blood vessel engineering. However, the tunable capacity of chitosan takes this biopolymer to applications beyond medicine. Thus, other challenges for the future are focused on the developing of nanocomposites for applications in biosensors, packaging, separation processes, the food or agricultural industry and catalytic processes. Another challenge is related to the valorization of chitosan into valuable organic compounds, which usually are obtained as alternatives to traditional fossil fuels.

One of the main challenges for the use of chitosan in the future must be the optimization of its degree of deacetylation using environmentally benign reagents. Another key parameter is the design of modulated tridimensional chitosan structures by crosslinking processes, which improve its use in specific applications. From a catalytic point of view, a main challenge is how to design porous chitosan-based catalysts to increase the amount of available active sites and thus improve the efficiency of catalytic processes.

## Figures and Tables

**Table 1:** Sources of chitin and chitosan.

Sea Animals	Insects	Microorganisms
Crustaceans	Scorpions	Green algae
Coelenterata	Brachiopods	Yeast (β-type)
Annelida	Cockroaches	Fungi (cell walls)
Mollusca	Spiders	Mycelia penicillium
Lobster	Beetles	Brown algae
Shrimp	Ants	Chytridiaceae
Prawn	-	Ascomydes
Krill	-	Blastocladiacease
Crab	-	Spores

**Table 2:** Several characterization methods to evaluate the deacetylation degree and average molecular weight of chitosan.

Characterization Methods	Chitosan Property	Ref.
Potenciometric titration	Deacetylation degree	[12,13]
Elemental analysis		[14]
Fourier transform infrared (FTIR)		[15,16,17,18]
Nuclear magnetic resonance (NMR)		[19,20]
Viscosimetry	Molecular weight	[21,22]
Gel permeation chromatography		[23,24,25]

**Table 4:** Some applications of chitosan in biomedical and pharmaceutical material.

● Treating major burns
● Preparation of artificial skin
● Surgical sutures
● Contact lenses
● Blood dialysis membranes
● Artificial blood vessels
● Antitumor
● Blood anticoagulant
● Antigastritis
● Haemostatic
● Hypochlesterolaemic agent
● Antithrombogeic agent
● Drug and gene-delivery systems
● Dental therapy

**Table 5:** Some applications of chitosan in tissue engineering.

● Cell growth and proliferation in tracheal cartilage, nerve
● Bone tissue repair and regeneration materials for cartilage repair
● Porous 3-D scaffold of chitosan-hydroxyapatite composites for bone regeneration
● Chitosan-chondroitin sulfate sponges in bone regeneration
● Chitosan-calcium alginate capsules to develop artificial pancreas for diabetes mellitus treatment

**Table 6:** Chitosan-based drug delivery systems.

Drug	Dosage Form
Aspirin	Wet granulation formulation
Chlorpheniramine maleate	Tablet
Dapsone	Gel
Oxyphenbutazone	Coated tablet
Prednisolone	Granules
Pullulan	Film

**Table 7:** Applications of chitosan in the field of food and nutrition.

Chitosan Application	Example
Additive	Clarification and deacilification of fruits and beverages
	Color stabilization
	Emusifying agent
	Food mimetic
	Natural flavor extender
	Texture controlling agent
	Thickening and stabilizing agent
Antimicrobial agent	Bactericidal
	Fungicidal
	Measure of mold contamination in agricultural commodities
Edible film industry	Controlled release of antimicrobial substances
	Controlled release of antioxidants
	Controlled release of nutrients, flavors and drugs
	Controlled moisture transfer between foo and surrounding environment
Nutritional quality	Antigastritis agent
	Dietary fiber
	Hypocholesterolemic effect
	Infant feed ingredient
	Livestock and fish feed additive
	Production of single cell protein

**Table 8:** Some microbial applications of chitosan and chitosan derivatives.

Chitosan/Chitosan Derivative	Microbial Strain	Application	Ref.
Chitosan	*Streptococcus*	Dental materials	[127]
	*Listeria monocytogenes, Pseudomonas aeruginosa* and *S. aureus*	Dairy food packaging	[128]
	*F. acuminatum, Cylindrocladium floridanum, Aspergillus flavus, Magnaporthe grisea, Bipolaris sorokiniana, F. graminearum, Phytophthora parasitica, Sclerotinia sclerotiorum*	Plant protection	[129]
Chitosan-polyphosphate-silver	*P. aeruginosa* and *S. aureus*	Wound dressing	[130]
Chitosan acetate	*P. aeruginosa, Proteus mirabilis* and *S. aureus*	Wound dressing	[131]
Carboxymethyl chitosan	*E. coli*	Fruit preservation	[132]
Chitosan-sulfonamide derivatives	*Staphylococcus aureus, Sarcina lutea, Bacillus cereus, Bacillus subtilis, Escherichia coli, Candida albicans, Candida glabrata and Candida sake*	Wound dressing and wound healing	[133,134]
*N*,*N*,*N*-trimethylchitosan polylactide/polypropylene fibers	*S. aureus*	Wound dressing	[135]
*N*-(Carboxymethyl) chitosan	*F. solani* and *C. lindemuthianum*	Plant protection	[129]
*N,N,N*-dimethylalkyl chitosans	*A. tumefaciens, E. carotovora, fungi B. cinerea, F. oxysporum,* and *P. debaryanum*	Crop protection	[136]
*N*-(*o,p*-Diethoxybenzyl)chitosan	*F. oxysporum* and *P. debaryanum*	Crop protection	[136]
*N*-(*o,o*-Dichlorobenzyl) chitosan, *N*-(*o,o*-dichloro-benzyl) chitosan, *N*,*O*-(*p*-chlorobutyryl) chitosan, *N*,*O*-decanoyl chitosan, *N*,*O*-cinnamoyl chitosan and *N*,*O*-(*p*-methoxy-benzoyl) chitosan	*B. cinerea*	Crop protection	[136]
*N*-Phenylalanine-*O*-carboxymethyl chitosan	*S. aureus* and *E. coli*	Food preservative coating	[137]
Chitosan/quaternary chitosan-polylactide	*S. aureus* and *E. coli*	Wound healing	[138]
Chitosan, chitosan-hydroxyapatite, *N*-[1-hydroxy-3-(trimethylammonium) propyl]chitosan chloride, carboxymethyl chitosan	*Streptococcus*	Dental care	[139,140]
*N*-[1-Hydroxy-3-(trimethylammonium) propyl]chitosan	*Bacillus subtilis*	Paper packaging	[141]
Carboxymethyl chitosan	*E. coli* and *S. aureus*	Cotton fabric	[142]
Poly(*n*-butyl acrylate)-chitosan	*S. aureus*	Cotton fabric	[143]
Chitosan-cellulose	*E. coli* and *S. aureus*	Membranes	[144]
*O*-Hydroxyethylchitosan-cellulose	*E. coli*	Textile	[145]
Chitosan-lauric acid-starch	*B. subtilis* and *E. coli*	Antimicrobial film	[123]
Dodecenyl succinylated phthaloyl chitosan	*E. coli*, *S. aureus* and *B. subtilis*	Antimicrobial film	[146]

**Table 9:** Chitosan-based composites used in the adsorption of dyes.

Adsorbent	Dye	Adsorption Capacity (mg g^−1^)	pH	Temperature (°C)	Ref.
Chitosan/activated clay	Methylene blue	330	7.1	30	[149]
	Reactive dye RR222	1912	6.5	30	
Chitosan/bentonite	Tartrazine	294	2.5	47	[150]
	Malachite green	435.0	6.0	37	[151]
Chitosan/kaolin/γ-Fe_2_O_3_	Methyl orange	-	6.0	-	[152]
Chitosan/montmorillonite	Congo red	53	7.0	30	[153]
Chitosan/oil palm	Reactive Blue 19	909	6.0	50	[154]
Chitosan/polyurethane	Acid violet 48	30	7.0	30	[155]

**Table 10:** Chitosan-based composites used in the adsorption of cations.

Adsorbent	Adsorbate	Maximum Adsorption Capacity (mg g^−1^)	pH	Temperature (°C)	Ref.
Chitosan/alginate	Cu^2+^	68	4.5	-	[161]
Chitosan/calcium arginate	Ni^2+^	222	5	-	[162]
Chitosan/cellulose	Cu^2+^	26	-	25	[163]
	Zn^2+^	20			
	Cr^6+^	13			
	Ni^2+^	13			
	Pb^2+^	26			
Chitosan/ceramic alumina	As^3+^	56	4.0	-	[164]
	As^5+^	96	4.0	25	
	Cu^2+^	86	-0-	-	[165]
	Ni^2+^	78	4	25	
	Cr^6+^	154	4	25	[166]
Chitosan/clinoptilolite	Cu^2+^	574	5.0	-	[167]
	Cu^2+^	719	5.0	25	[168]
	Co^2+^	468			
	Ni^2+^	247			
Chitosan/cotton fibers (via C-N single bond)	Hg^2+^	96	5.0	25	[169]
	Au^3+^	89	3.0	25	[170]
Chitosan/cotton fibers (via Schiff base bon)	Hg^2+^	104	5.0	35	[169]
	Au^3+^	77	3.0	25	[170]
	Cu^2+^	25	6.5	25	[171]
	Ni^2+^	8			
	Pd^2+^	102			
	Cd^2+^	16			
Chitosan/magnetite	Cr^6+^	69	4.0	-	[172]
	Pb^2+^	63	6.0	-	[173]
	Ni^2+^	53			
Chitosan/perlite	Cu^2+^	196	5.0	-	[174]
	Ni^2+^	115			
	Cd^2+^	179	6.0	25	[175]
	Cr^6+^	154	4.0	25	[176]
	Cu^2+^	104	4.5	25	[177]
Chitosan/polyvinyl alcohol	Cd^2+^	143	6.0	50	[178]
	Cu^2+^	48	6.0	-	[179]
Chitosan/polyvinyl chloride	Cu^2+^	88	4.0	-	[180]
	Ni^2+^	120	5.0		
Chitosan/silica	Ni^2+^	254	5.0	-	[161]

**Table 11:** Chitosan-based composites and its uses in separation processes.

Application	Membrane	Ref.
Water/ethanol mixture separation	Chitosan salt	[183]
	Crosslinked chitosan	[184]
	Chitosan/*N*-methyol nylon 6 blend	[185]
	HY zeolite-filled chitosan	[186]
	Crossline quaternized chitosan composite	[187]
	Chitosan-hydroxyethylcellulose composite	[182]
Isopropanol-water separation	Chitosan	[188]
	Chitosan-hydroxyethylcellulose blended	[189]
	Crosslinked chitosan	[190]
	Chitosan/NaY zeolite composite	[191]
	Blended chitosan/polyvinyl alcohol	[192]
	Chitosan-poly(tetrafluoroethylene) composite	[193]
	Crosslinked carboxymethyl chitosan-PSF-hollow-fiber composite	[194]
	Diisocyanate crosslinked chitosan	[195]
	Chitosan-polyacrylonitrile hollow fiber	[196]
	Poyelectrolyte complexes of chitosan and phosphotungstic acid	[197]
	Chitosan g-polyaniline	[198]
	Sodium alginate and chitosan-wrapped MWCNT	[199]
Ethylene glycol/H_2_O separation	Surface crosslinked chitosan	[200]
	Chitosan-poly(acrylic acid) polyelectrolyte complex	[201]
	Chitosan polysulfone composite	[202]
	Chitosan poly(vinyl alcohol) blend	[203]
Separation methanol/methyl *t*-butyl ether	Chitosan-poly (N.vinyl-2-pyrrolidone) blend	[204]
	Chitosan composite (modified with surfactants)	[205]
	Chitosan-anionic surfactant complex	[206]
Separation alcohol-toluene	*N*-acetylated chitosan	[207]
	Silicate zeolite embedded chitosan mixed matrix	[208]
Separation dimethyl carbonate-methanol	Chitosan	[209]
	ZSM-5 zeolite-filled chitosan	[210]
Separation benzene-cyclohexane	Poly(vinyl alcohol) chitosan blend	[211]
	Chitosan/Ag^+^-carbon nanotubes	[212]
Dehydration of 1,4-dioxane	Poly(vinyl alcohol)/chitosan	[213]
	Chitosan/nylon 66	[214]
	Crosslinked calcium alchinate-chitosan blend	[215]
	Poly(3-hydroxybutyrate)-functionalized multiwalled carbon nanotubes-chitosan composite	[216]
Dehydration of caprolactam	Crosslinked PVA/chitosan	[217]
	Chitosan-konjac glucomannan blending	[218]
	Chitosan-poly(acrylic acid) composite	[219]
