# Correction: Jiménez-Gómez, C.P.; Cecilia, J.A. Chitosan: A Natural Biopolymer with a Wide and Varied Range of Applications. *Molecules* 2020, *25*, 3981

**DOI:** 10.3390/molecules31193390

**Published:** 2026-09-24

**Authors:** Carmen P. Jiménez-Gómez, Juan Antonio Cecilia

**Affiliations:** Departamento de Química Inorgánica, Cristalografía y Mineralogía (Unidad Asociada al ICP-CSIC), Facultad de Ciencias, Universidad de Málaga, Campus de Teatinos, 29071 Malaga, Spain; carmenpjg@uma.es

## Error in Figure

In the original publication [1], there was a mistake in Figure 3 as published. Figure 3 is missing the protonated amine group. The corrected Figure 3 appears below. The authors state that the scientific conclusions are unaffected. This correction was approved by the Academic Editor. The original publication has also been updated.

## Figures and Tables

**Figure 3:**
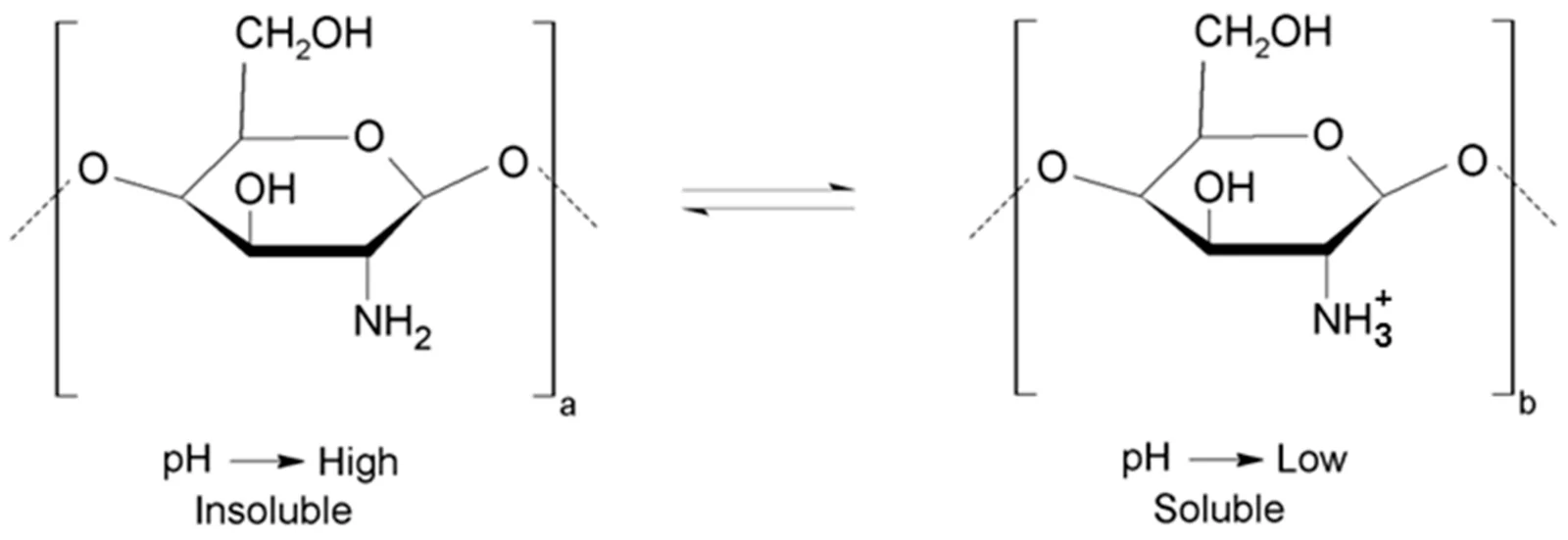
Chemical structure of chitosan as a function of pH. Insoluble (pH > 6) and soluble (pH < 6).
